# Decomposition of Molecular Charge and Spin Transfer Global Indexes into Atomic Group Contributions

**DOI:** 10.1021/acs.jctc.5c02145

**Published:** 2026-04-03

**Authors:** Carlo Gatti, Yann Danten, Christine Frayret

**Affiliations:** † 9327 CNR SCITEC, CNR Istituto di Scienze e Tecnologie Chimiche ‘Giulio Natta’’, Sede Via C. Golgi, 19, 20133 Milano, Italy; ‡ Istituto Lombardo, Accademia di Scienze e Lettere, via Brera 76, 20133 Milano, Italy; § Institut des Sciences Moléculaires, UMR CNRS 5255, 351 Cours de la Libération, 33405 Talence, France; ∥ 131891 Laboratoire de Réactivité et Chimie des Solides (LRCS), UMR CNRS 7314, Université de Picardie Jules Verne, Hub de l’Energie, 15 Rue Baudelocque, 80000 Amiens Cedex, France; ⊥ Réseau sur le Stockage Electrochimique de l’Energie (RS2E), FR CNRS 3459, 80039 Amiens Cedex, France

## Abstract

We recently introduced a model for decomposing the global charge transfer (CT) excitation indexes proposed by Le Bahers, Adamo, and Ciofini ( Le BahersT.,  J. Chem. Theory Comput. 2011, 7, 2498–2506 26606624 10.1021/ct200308m) into contributions from molecular subdomains ( GattiC.,  J. Phys. Chem. A 2022, 126, 6314–6328 36053727 10.1021/acs.jpca.2c04607PMC9483980), together with a software tool, DOCTRINE (atomic group *Decomposition Of the Charge TRansfer INdExes*), which implements this approach. DOCTRINE has been successfully applied to several excited states (ESs) of a push–pull compound in different solvent environments. In this work, we extend our previous model to spin-polarized systems by introducing, in addition to the global CT excitation indexes, their analogous electron spin transfer (ST) indexes. These can also be decomposed into chemically significant contributions from molecular subdomains. This extension provides a set of related CT and ST descriptors, enabling a visual and quantitative differentiation of the behavior of electronic charge and spin transfers. The updated DOCTRINE_SPIN version of the software now includes computation of ST indexes and their associated descriptors, broadening the applicability of the method to spin-resolved electronic excitations. Our CT and ST decomposition model is applicable to any partitioning of real space, whether fuzzy or disjoint and exhaustive. However, we apply it in terms of chemically relevant molecular subdomains based on the Atoms in Molecules (AIM) Bader’s basins, taking advantage of associating *intra*- and *inter*-subdomain contributions with rigorously defined quantum objects that retain clear chemical meaning. The model allows for a quantitative evaluation of subdomain contributions to the CT, the ST, and their excitation lengths, and to the charge- and spin-transfer dipole moments. Although these global indexes can be derived either from electron and spin density increments or from their depletions upon excitation, the subdomain contributions obtained from the two distributions generally differ. This distinction helps to determine whether a given property’s contribution from a subdomain is dominated by one of the distributions or whether both play a significant role. As an initial application of our spin-polarized model extension, we selected a π-conjugated (acceptor–donor–acceptor) compound (TMTQ), composed of a central 1,6-methano[10]­annulene (M10A) and 5-dicyanomethyl-thiophene (DT) peripheries in an exo geometry. TMTQ exhibits a singlet–triplet energy gap of only 4.9 kcal/mol, with the singlet state being more stable than the triplet. This small energy gap arises from the different weights of nearly degenerate mesomeric structures with distinct electron delocalization patterns. The electronic charge (and spin) transfers occurring upon excitation of the singlet and triplet ground states (GS) (S_0_ and T_1_) to their first five excited states (S_1_–S_5_ and T_2_–T_6_) are characterized and compared, highlighting their distinct features, the role of ST on CT when both transfers are possible, and the resulting effects on electron and spin delocalizations.

## Graphical abstract

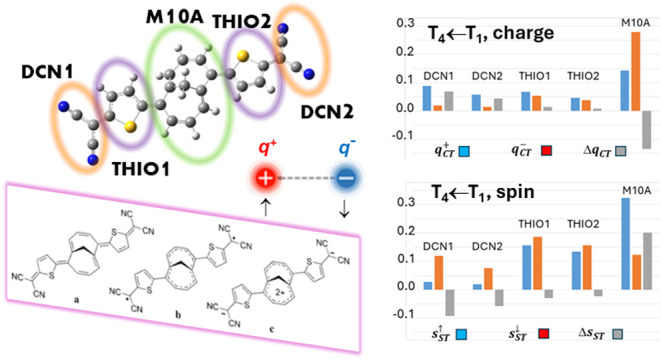

## Introduction

A proper assessment and a deep understanding of the properties of electronically excited states is a challenging task, but one that is becoming increasingly central to many fields, including chemistry, molecular physics, molecular biology, spectroscopy, and materials science. The computation of the electronic structures for excited states has made significant progress over the last decade, not only due to the continuous growth of computational power but also thanks to the novel and increasingly powerful electronic structure methods that have become progressively available.^1–5^ A major impetus for this progress has been the remarkable advances^2^ in time-resolved spectroscopies, which have led to a growing demand for theoretical methods capable of accurately describing the characteristic features of excited states. The ability to perform detailed computations of electron transitions and evaluate the excited state wavefunctions and properties for increasingly larger molecular systems has, in parallel, driven substantial efforts to develop methods capable of analyzing and understanding the nature of excited state electronic structures in depth. As the size of the systems increases, this electronic structure becomes more complex, often revealing new and qualitatively distinct physical behaviors.

Methods for automating the analysis of excited states, supported by visualization techniques^6–8^ and aimed at providing rigorous and reproducible descriptors for measuring charge transfer (CT),^9–14^ double excitation character,^8,15–17^ entanglement,^18–21^ and, more generally, at revealing phenomena that are not captured by the standard molecular orbital (MO) picture,^12,22,23^ have been described. Among these approaches, TheoDORE^22,23^ stands out as one of the most successful and user-friendly toolboxes in the field. Its distributed software code is interfaced with ten different quantum chemistry codes and a range of excited state methods implemented within it. Three powerful functionalities of TheoDORE have gained special popularity: a “fragment-based analysis for assigning state character, the computation of exciton sizes for measuring charge transfer, and the use of natural transition orbitals not only for visualization but also for quantifying multiconfigurational character”.^22^

In a recent paper,^24^ we introduced a novel method for rigorously decomposing CT excitation descriptors (also called CT global indexes) into the contributions of atomic groups (or molecular subdomains). Our method generalizes the CT global indices model, developed a few years ago by Le Bahers, Adamo, and Ciofini (hereafter referred to as the LBAC model).^10^ The CT global indices in the LBAC model are all defined based solely on the ground and excited state electron densities (EDs) of the system. The barycentres or centroids of the ED depletion and enrichment regions upon electron transition are evaluated, and the CT length is simply the distance between these centroids. The transferred charge is obtained by integrating either the ED depletion or enrichment distribution over the entire molecular space, and the product of the CT length and the transferred charge gives the change in the dipole moment upon electron transition. This dipole moment change can then be compared with that obtained from the computed *ab initio* ground state and excited state wavefunctions to verify the accuracy of the adopted integration procedures.

Our proposed method^24^ allows for decomposing all the key quantities of the LBAC model into the contribution of molecular moieties, where the moieties are defined in terms of either a fuzzy or a disjoint exhaustive partitioning of real space. Although both partitioning methods are possible, we preferentially selected the disjoint exhaustive partitioning provided by Bader’s atomic basins^25^ for the initial applications^24,26^ of our method. We characterized the significant CT occurring by exciting the ground state of a push-pull compound to its first four singlet excited states in several solvents with increasing polarity. Push-pull systems, consisting of an electron donor (D) and of a covalently connected electron-withdrawing or -acceptor (A) group have been extensively studied due to their intense, solvatochromic, optical transitions.^10,11,23,27–30^ However, the simple mechanism for the formation of an excited state, where an electron is transferred from D to A to form a [*D* ^+^ *A* ^–^]* excited state, represents only an idealized situation.^31^ In reality, excitations may be either local or delocalized in character, and they may not necessarily occur from the D to A. Furthermore, the effective charge transferred may be much lower than the ideal value of one for single-electron excitation processes.^22,30–32^ By selecting chemically meaningful moieties as subdomains, we were able to effectively address such a complex scenario, gaining clear chemical insights into the CT process for the various excited states and highlighting the role of solvent polarity modulation.

Photoinduced electron-transfer (PET) is a key mechanism in various chemical, physical, and biological processes, all of which have been extensively studied for their applications, including light-to-chemical energy conversion, molecular photoelectronics, photocatalysis, and photosynthesis. The ability to tune and/or improve CT, as well as identify the key actors involved in electron-transfer mechanisms, is clearly a prerequisite for designing optimized systems. The extension of the LBAC model to a subdomain representation enabled us to distinguish and quantify the local intra-subdomain effects from the synergistic or anti-synergistic coupled effects of all subdomain pairs on the global CT indices, thus providing further valuable insights into PET processes.

Our extension of the LBAC model bears some resemblance to the fragment-based analysis in TheoDORE,^22,23^ which is capable of identifying the system fragments that contribute most to a given excitation, as well as the portions of the molecule where CT predominantly occurs. However, the hole–electron decomposition is performed by TheoDORE in Hilbert space, whereas in our method it is carried out in real space. In TheoDORE, the CT analysis involves the one-electron transition density matrix, while both the LBAC model^10^ and its generalization to subdomains use the *rearranged* electron density (ED), specifically the difference between the excited and ground state (GS) EDs. The charge transfer “numbers”^22^ within a fragment and between fragments are calculated in THEDORE using population analysis schemes, which may, therefore, be influenced by basis set and computational method dependence. In contrast, the fact that our model evaluates contributions in the subdomain real space ensures that the intra- and inter-fragments contributions are much less dependent on the basis set and computational method, provided the chosen basis set and method are of sufficient quality.

In this work, we extend our previous model to spin-polarized systems by introducing electron spin transfer (ST) indexes, in addition to the global Charge Transfer (CT) excitation indexes. Like the CT indexes, the ST indexes can be decomposed into chemically meaningful contributions from molecular subdomains. Although the ST decomposition model can, in principle, be applied to any real-space partitioning, we adopt a decomposition based on chemically relevant molecular subdomains defined by Bader’s Atoms in Molecules (AIM) basins,^25^ for the same reasons as in the CT case. This extension provides a set of related CT and ST descriptors, enabling a visual and quantitative differentiation of the behavior of electronic charge and spin transfers. In particular, the model allows for a quantitative evaluation of subdomain contributions to the CT, the ST, and their excitation lengths and to the charge- and spin-transfer dipole moments. Although these global indexes can be derived either from electron and spin density increments or from their depletions upon excitation, the subdomain contributions obtained from the two distributions generally differ. This distinction helps to determine whether a given property’s contribution from a subdomain is dominated by one of the distributions or whether both play a significant role.

As an initial application of our spin-polarized model extension, we selected a π-conjugated (acceptor-donor-acceptor or A–D–A) compound (TMTQ), composed of a central 1,6-methano[10]­annulene (M10A) and 5-dicyanomethyl-thiophene (DT) peripheries in an exo geometry.^33,34^ TMTQ exhibits a singlet–triplet energy gap of only 4.9 kcal/mol, with the singlet state being more stable than the triplet.^33^ This small energy gap arises from the different weights of nearly degenerate mesomeric structures with distinct electron delocalization patterns.^33,34^ The electronic charge (and spin) transfers occurring upon excitation of the singlet and triplet ground states (S_0_ and T_1_) to their first five excited states (S_1_–S_5_ and T_2_–T_6_) are characterized and compared, highlighting their distinct features, the role of ST on CT when both transfers are possible, and the resulting effects on electron and spin delocalizations.

## Theory and Computational Details

The LBAC model^10^ is summarized below to present its recently proposed atomic group decomposition version and introduce an extension of this version capable of treating spin transfer accompanying any excitation between spin-polarized states or between states with different total spin (one of the two states can be, but not necessarily, spin-unpolarized). The newly proposed spin transfer atomic group decomposition model is outlined, and details of its practical implementation are provided. Computational details for the application of the developed method to the TMTQ system are then presented.

### A Quantitative Index of the Spatial Extent of Charge Transfer (CT) and Spin Transfer (ST) Excitations: The LBAC Model and Its Extension to Electron Spin Density Changes

In the LBAC model, excitations between the ground state (GS) and one of its vertical excited states (ES) *n* are analyzed in terms of ρ_GS_ and ρ_ES,*n*_, the electronic densities of the ground state (GS) and the vertical excited state *n*, respectively. The electron density (ED) rearrangement due to the electronic transition *Y*_*n*_ ← *Y*_0_ is given by:

1 $$\Delta \rho \left(r;Y_{n}\leftarrow Y_{0}\right)=\rho_{ES,n}\left(r\right)-\rho_{GS}\left(r\right)$$

where Δρ denotes a local increment (Δρ > 0) or a local depletion (Δρ < 0) of the ED upon electronic transition, and where Δρ integrates to zero over the entire space *R* ^3^ (for the sake of simplicity, Δρ­(***r***; *Y*_*n*_ ← *Y*_0_) will hereafter be written simply as Δρ­(***r***)). By defining ρ^+^(***r***) and ρ^–^(***r***) as follows,

2 $$\begin{array}{l} \rho^{+}\left(r\right)=\Delta \rho \left(r\right)\;\mathrm{if}\;\Delta \rho \left(r\right)>0,\;\mathrm{otherwise}\;\rho^{+}\left(r\right)=0\\ \rho^{-}\left(r\right)=\left|\Delta \rho \left(r\right)\right|\;\mathrm{if}\;\Delta \rho \left(r\right)<0,\;\mathrm{otherwise}\;\rho^{-}\left(r\right)=0 \end{array}$$

it is easy to obtain the amount of transferred charge *q*_CT_ upon excitation as the following quantity:

3 $$q_{CT}\left(Y_{n}\leftarrow Y_{0}\right)=\int_{R^{3}}\rho^{+}\left(r\right)dr\equiv \int_{R^{3}}\rho^{-}\left(r\right)dr$$

The value of *q*_CT_ will range between 0 and 1 for a one-electron excitation, being close to 1 for an almost ideal one-electron transfer, and much smaller in real situations.

If the excitation involves an electron spin density (SD) change, we can similarly define the electron SD rearrangement due to the electronic/spin transition *Y*_*n*_ ← *Y*_0_ as

4 $$\Delta s\left(r;Y_{n}\leftarrow Y_{0}\right)=s_{ES,n}\left(r\right)-s_{GS}\left(r\right)$$

where *s*_GS_ and *s*_ES,*n*_ are the electronic spin densities of the ground state (GS) *Y*_0_ and of its vertical excited state *Y*_*n*_, respectively (note that our method applies to any vertical excitation in which at least one of the two involved states has a nonzero spin quantum number *S*. Therefore, eq 4 also covers the special case where the spin density of one of the two states is zero everywhere). In eq 4, Δ*s* denotes a local increment (Δ*s* > 0) or a local depletion (Δ*s* < 0) of the SD upon the electronic/spin transition and Δ*s* integrates, over the entire space *R* ^3^, to the difference in the number of unpaired electrons between the two involved states. If the states are characterized by the same quantum spin *S* (for instance, a transition from the triplet GS, T_1_, to one of its excited triplet states, T_*n*_), then Δ*s* integrates to zero over the entire space *R* ^3^. Analogous to the definition of Δρ, we can define the two scalar distributions *s* ^+^(***r***) and *s* ^–^(***r***) as follows:

5 $$\begin{array}{l} s^{+}\left(r\right)=\Delta s\left(r\right)\;\mathrm{if}\;\Delta s\left(r\right)>0,\;\mathrm{otherwise}\;s^{+}\left(r\right)\;=0\\ s^{-}\left(r\right)=\left|\Delta s\left(r\right)\right|\;\mathrm{if}\;\Delta s\left(r\right)<0,\;\mathrm{otherwise}\;s^{-}\left(r\right)=0 \end{array}$$

From these distributions, one can determine the amount of spin-up electron transferred, *s*_ST_^↑^, and spin-down electron transferred, *s*_ST_^*↓*^, upon excitation:

6 $$\begin{array}{l} s_{\mathrm{ST}}^{↑}\left(Y_{n}\leftarrow Y_{0}\right)=\int_{R^{3}}s^{+}\left(r\right)dr\\ s_{\mathrm{ST}}^{↓}\left(Y_{n}\leftarrow Y_{0}\right)=\int_{R^{3}}s^{-}\left(r\right)dr \end{array}$$

where *s*_ST_^↑^(*Y*_*n*_ ← *Y*_0_) and *s*_ST_^↓^(*Y*_*n*_ ← *Y*_0_) are equal in value if *Y*_*n*_ and *Y*_0_ have the same quantum spin *S.* Therefore, in this case, their difference Δ*s*_ST_(*Y*_*n*_ ← *Y*_0_) is zero:

7 $$\Delta s_{\mathrm{ST}}\left(Y_{n}\leftarrow Y_{0}\right)=s_{\mathrm{ST}}^{↑}\left(Y_{n}\leftarrow Y_{0}\right)-s_{\mathrm{ST}}^{↓}\left(Y_{n}\leftarrow Y_{0}\right)$$

However, if the two states differ in their quantum spin *S*, this difference will correspond to the difference in the number of their unpaired electrons. When these states have the same quantum spin *S*, we will denote both *s*_ST_^↑^ and *s*_ST_^↓^, whenever possible, simply as *s*_ST_, since the two quantities are then equal. In contrast to *q*_CT_, and regardless of whether the two states have the same or different quantum spin *S*, there is no upper bound for *s*_ST_ (except that determined by the total number of electrons in the system). Indeed, *s*_ST_ can exceed 1 for a one-electron excitation.

The centroids, or the positions of the poles of the positive and negative transferred charges, are given by

8 $$\begin{array}{l} R^{+}\left(Y_{n}\leftarrow Y_{0}\right)=\frac{\int_{R^{3}}r\rho^{+}\left(r\right)dr}{\int_{R^{3}}\rho^{+}\left(r\right)dr}\\ R^{-}\left(Y_{n}\leftarrow Y_{0}\right)=\frac{\int_{R^{3}}r\rho^{-}\left(r\right)dr}{\int_{R^{3}}\rho^{-}\left(r\right)dr} \end{array}$$

Accordingly, a measure of the CT excitation length, *D*_CT_, is obtained from:

9 $$D_{\mathrm{CT}}\left(Y_{n}\leftarrow Y_{0}\right)=\left|R^{+}-R^{-}\right|$$

The norm of the change in dipole moment from the ground to excited states, **μ**_CT_, is calculated as:

10 $$||\mu_{\mathrm{CT}}||\left(Y_{n}\leftarrow Y_{0}\right)=D_{\mathrm{CT}}\left(Y_{n}\leftarrow Y_{0}\right)\cdot q_{\mathrm{CT}}\left(Y_{n}\leftarrow Y_{0}\right)$$

Finally, the deviation of this norm from the difference between the *ab initio* dipole moment magnitudes calculated for the ground and excited state *n* provides an estimate of the integration accuracy in eqs 3 and 8 (see *infra*).

For a vertical excitation between spin-polarized states with the same quantum spin *S*, we can define the centroids, or the positions of the poles of the spin-up and spin-down transferred spins, in perfect analogy with those of the transferred charge. These are given by:

11 $$\begin{array}{l} S^{↑}\left(Y_{n}\leftarrow Y_{0}\right)=\frac{\int_{R^{3}}rs^{+}\left(r\right)dr}{\int_{R^{3}}s^{+}\left(r\right)dr}\\ S^{↓}\left(Y_{n}\leftarrow Y_{0}\right)=\frac{\int_{R^{3}}rs^{-}\left(r\right)dr}{\int_{R^{3}}s^{-}\left(r\right)dr} \end{array}$$

Accordingly, a measure of the spin-up and spin-down transfer (ST) excitation length, *D*_ST_, is obtained from:

12 $$D_{\mathrm{ST}}\left(Y_{n}\leftarrow Y_{0}\right)=\left|S^{↑}-S^{↓}\right|$$

The norm of the spin density dipole moment caused by the spin-up and spin-down transfer, **μ**_ST_, is defined as:

13 $$||\mu_{\mathrm{ST}}||\left(Y_{n}\leftarrow Y_{0}\right)=D_{\mathrm{ST}}\left(Y_{n}\leftarrow Y_{0}\right)\cdot s_{\mathrm{ST}}\left(Y_{n}\leftarrow Y_{0}\right)$$

In general, ∥**μ**_ST_∥ will differ from ∥**μ**_CT_∥ because the separation *D*_ST_ between the spin-up and spin-down poles of the transferred spin, as well as the amount of the spin-up and spin-down transferred spin, will generally differ from the corresponding charge transfer counterparts.

The differences between ∥**μ**_ST_∥ and ∥**μ**_CT_∥, and between their respective charge/spin transfers and lengths, provide detailed insight into how charge and spin rearrangements occur differently during a given excitation.

For a vertical excitation between states with different quantum spin *S*, the concept of a spin density dipole is no longer applicable. However, the centroids of the spin-up and spin-down rearrangements due to the excitation, as well as their distance, can still be evaluated using eqs 11 and 12.

Before concluding this paragraph, it is important to note that in eq 1 (and eq 4), we use a sign convention opposite to the one used in our previous paper, but consistent with that adopted in the original LBAC model. Both sign conventions are valid and correct. Our previous choice had the advantage of associating positive Δρ values with regions that, upon transition to the excited state, decrease their electron concentration (and thus become *positively charged* relative to the initial state), while negative Δρ values corresponded to regions where electron concentration increases (becoming *negatively charged* relative to the initial state). The current convention, however, has the advantage of aligning with the commonly adopted sign convention in the literature, which helps to avoid potential confusion for the reader. Additionally, in the case of spin density distributions, it assigns positive values to regions where the α-spin character is enhanced upon excitation relative to the initial state (Δ*s* > 0). Since polarized states are generally assumed to have an excess of total α-spin electrons, considering regions that increase their α-spin density upon excitation as positive appears more intuitive than the opposite convention.

### Atomic Group Decomposition of Charge and Spin Transfer Global Indexes

By assuming either a fuzzy or a disjoint exhaustive partitioning of *R* ^3^ into subdomains Ω, eq 3 may be rewritten as:

14 $$q_{\mathrm{CT}}\left(Y_{n}\leftarrow Y_{0}\right)=\sum_{\Omega }\int_{\Omega }\rho^{+}\left(r\right)dr\equiv \sum_{\Omega }\int_{\Omega }\rho^{-}\left(r\right)dr$$

This formulation allows us to interpret *q*_CT_ as the result of contributions from each subdomain. Similarly, a corresponding decomposition into subdomain contributions can be applied to the changes in spin density distribution upon excitation. If the two states *Y*_*n*_ and *Y*_0_ have the same quantum spin *S*, eq 6 can be expressed as:

15 $$s_{\mathrm{ST}}\left(Y_{n}\leftarrow Y_{0}\right)=\sum_{\Omega }\int_{\Omega }s^{+}\left(r\right)dr\equiv \sum_{\Omega }\int_{\Omega }s^{-}\left(r\right)dr$$

As previously noted for the charge transfer decomposition,^24^ the equivalence of the sums of subdomain contributions calculated by integrating either ρ^+^(***r***) or ρ^–^(***r***) (or similarly *s* ^+^(***r***) or *s* ^–^(***r***)) over the entire molecular system does not hold for the individual subdomain contributions. In general, the following inequalities hold:

16 $$\int_{\Omega }\rho^{+}\left(r\right)dr\neq \int_{\Omega }\rho^{-}\left(r\right)dr,\left(\mathrm{or}\;\int_{\Omega }s^{+}\left(r\right)dr\neq \int_{\Omega }s^{-}\left(r\right)dr\right)$$

Thus, the following subdomain quantities are defined as:

17a $$q_{\mathrm{CT}}^{+}\left(\Omega ,Y_{n}\leftarrow Y_{0}\right)=\int_{\Omega }\rho^{+}\left(r\right)dr$$

17b $$q_{\mathrm{CT}}^{-}\left(\Omega ,Y_{n}\leftarrow Y_{0}\right)=\int_{\Omega }\rho^{-}\left(r\right)dr$$

17c $$\Delta q_{\mathrm{CT}}\left(\Omega ,Y_{n}\leftarrow Y_{0}\right)=q_{\mathrm{CT}}^{+}\left(\Omega ,Y_{n}\leftarrow Y_{0}\right)-q_{\mathrm{CT}}^{-}\left(\Omega ,Y_{n}\leftarrow Y_{0}\right)$$

and

18a $$s_{\mathrm{ST}}^{↑}\left(\Omega ,Y_{n}\leftarrow Y_{0}\right)=\int_{\Omega }s^{+}\left(r\right)dr$$

18b $$s_{\mathrm{ST}}^{↓}\left(\Omega ,Y_{n}\leftarrow Y_{0}\right)=\int_{\Omega }s^{-}\left(r\right)dr$$

18c $$\Delta s_{\mathrm{ST}}\left(\Omega ,Y_{n}\leftarrow Y_{0}\right)=s_{\mathrm{ST}}^{↑}\left(\Omega ,Y_{n}\leftarrow Y_{0}\right)-s_{\mathrm{ST}}^{↓}\left(\Omega ,Y_{n}\leftarrow Y_{0}\right)$$

Equations 17–19 and 20–22 are crucial. They indicate that a subdomain can have an ambivalent nature, with some regions contributing to the positive and others to the negative poles of the transferred charge (eqs 17–18), or with some regions contributing to the spin-up and others to the spin-down poles of the transferred spin (eqs 20–21). These contributions are generally unequal in magnitude (and, in some cases, significantly so), and the regions acting as sources for either the positive (spin-up) or negative (spin-down) poles are typically quite distinct for charge (and spin-up/spin-down) transfers. The quantities Δ*q*_CT_(Ω) and Δs_ST_(Ω) (eqs 19 and 22) allow us to evaluate whether a subdomain is more efficient at creating one or the other of the two poles of charge or spin rearrangements upon excitation. Since ∑_Ω_Δ*q*_CT_(Ω, *Y*_*n*_ ← *Y*_0_) = 0 and ∑_Ω_Δ*s*_ST_(Ω, *Y*_*n*_ ← *Y*_0_) = 0 (for excitations between states with same quantum spin *S*), the following inequalities hold:

19a $$A=\sum_{\Omega ,\Delta q_{\mathrm{CT}}>0\;}\Delta q_{\mathrm{CT}}\left(\Omega ,Y_{n}\leftarrow Y_{0}\right)\leq q_{\mathrm{CT}}\left(Y_{n}\leftarrow Y_{0}\right)$$

19b $$A=\sum_{\Omega ,\Delta q_{\mathrm{CT}}<0\;}\left|\Delta q_{\mathrm{CT}}\left(\Omega ,Y_{n}\leftarrow Y_{0}\right)\right|\leq q_{\mathrm{CT}}\left(Y_{n}\leftarrow Y_{0}\right)$$

19c $$B=\sum_{\Omega ,\Delta s_{\mathrm{ST}}>0}\Delta s_{\mathrm{ST}}\left(\Omega ,Y_{n}\leftarrow Y_{0}\right)\leq s_{\mathrm{ST}}\left(Y_{n}\leftarrow Y_{0}\right)$$

19d $$B=\sum_{\Omega ,\Delta s_{\mathrm{ST}}<0}\left|\Delta s_{\mathrm{ST}}\left(\Omega ,Y_{n}\leftarrow Y_{0}\right)\right|\leq s_{\mathrm{ST}}\left(Y_{n}\leftarrow Y_{0}\right)$$

The equality in eqs 23–24 ([Disp-formula eq25]–[Disp-formula eq26]) is only achieved in the rare case where a subdomain has either only positive or only negative Δρ­(***r***) (Δ*s*(***r***)) values.

Similar to *q*_CT_ and *s*_ST_, *D*_CT_ and *D*_ST_ can also be decomposed in terms of subdomain contributions:

20a $$D_{\mathrm{CT}}\left(Y_{n}\leftarrow Y_{0}\right)=\;\left|R^{+}-R^{-}\right|=\left|\frac{\int_{R^{3}}r\rho^{+}\left(r\right)dr}{\int_{R^{3}}\rho^{+}\left(r\right)dr}-\frac{\int_{R^{3}}r\rho^{-}\left(r\right)dr}{\int_{R^{3}}\rho^{-}\left(r\right)dr}\right|=\left|\frac{\sum_{\Omega }\int_{\Omega }r\rho^{+}\left(r\right)dr}{q_{\mathrm{CT}}}-\frac{\sum_{\Omega }\int_{\Omega }r\rho^{-}\left(r\right)dr}{q_{\mathrm{CT}}}\right|=\left|\frac{\sum_{\Omega }\int_{\Omega }r\left[(\rho^{+}\left(r\right)-\rho^{-}\left(r\right)\right]dr}{q_{\mathrm{CT}}}\right|=\left|\sum_{\Omega }d_{\mathrm{CT}}^{\Omega }\left(Y_{n}\leftarrow Y_{0}\right)\right|$$

20b $$D_{\mathrm{ST}}\left(Y_{n}\leftarrow Y_{0}\right)=\left|S^{↑}-S^{↓}\right|=\left|\frac{\int_{R^{3}}rs^{+}\left(r\right)dr}{\int_{R^{3}}s^{+}\left(r\right)dr}-\frac{\int_{R^{3}}rs^{-}\left(r\right)dr}{\int_{R^{3}}s^{-}\left(r\right)dr}\right|=\left|\frac{\sum_{\Omega }\int_{\Omega }rs^{+}\left(r\right)dr}{s_{\mathrm{ST}}}-\frac{\sum_{\Omega }\int_{\Omega }rs^{-}\left(r\right)dr}{s_{\mathrm{ST}}}\right|=\left|\frac{\sum_{\Omega }\int_{\Omega }r\left[(s^{+}\left(r\right)-s^{-}\left(r\right)\right]dr}{s_{\mathrm{ST}}}\right|=\left|\sum_{\Omega }d_{\mathrm{ST}}^{\Omega }\left(Y_{n}\leftarrow Y_{0}\right)\right|$$

where **d**_CT_^Ω^ and **d**_ST_^Ω^, unlike *D*_CT_ and *D*_ST_, are 3-component vectors. It may be convenient to introduce the corresponding vectors for the entire system, **d**_CT_, and **d**_ST_, whose components are given by a sum over the corresponding subdomain components:

21a $$d_{\mathrm{CT},j}={\left(R^{+}-R^{-}\right)}_{j}=\sum_{\Omega }d_{\mathrm{CT},j}^{\Omega }\left(Y_{n}\leftarrow Y_{0}\right),j=x,y,z$$

21b $$d_{\mathrm{ST},j}={\left(S^{↑}-S^{↓}\right)}_{j}=\sum_{\Omega }d_{\mathrm{ST},j}^{\Omega }\left(Y_{n}\leftarrow Y_{0}\right),j=x,y,z$$

While

22a $$g_{\mathrm{CT},j}^{\Omega }\left(Y_{n}\leftarrow Y_{0}\right)=\frac{d_{\mathrm{CT},j}^{\Omega }\left(Y_{n}\leftarrow Y_{0}\right)}{D_{\mathrm{CT}}\left(Y_{n}\leftarrow Y_{0}\right)}$$

22b $$g_{\mathrm{ST},j}^{\Omega }\left(Y_{n}\leftarrow Y_{0}\right)=\frac{d_{\mathrm{ST},j}^{\Omega }\left(Y_{n}\leftarrow Y_{0}\right)}{D_{\mathrm{ST}}\left(Y_{n}\leftarrow Y_{0}\right)}$$

are dimensionless quantities that provide a measure of the lengths of **d**_CT, j_^Ω^(*Y*_*n*_ ← *Y*_0_) or **d**_ST, j_^Ω^(*Y*_*n*_ ← *Y*_0_) relative to *D*_CT_ or *D*_ST_, respectively. Negative signs for ***g***_CT,*j*_^Ω^ or ***g***_ST,*j*_^Ω^ indicate that **d**_CT, j_^Ω^ or **d**_ST, j_^Ω^ are oppositely directed to (**R** ^+^ – **R** ^–^)_j_ or (**S** ^↑^ – **S** ^↓^)_j_, respectively. The direction cosines of (**R** ^+^ – **R** ^–^) or (**S** ^↑^ – **S** ^↓^), given by

23a $$\alpha_{\mathrm{CT},j}\;\left(j=x,y,z\right)=\cos a_{\mathrm{CT},j}=\frac{{\left(R^{+}-R^{-}\right)}_{j}}{\left|R^{+}-R^{-}\right|}=\frac{d_{\mathrm{CT},j}}{D_{\mathrm{CT}}}$$

23b $$\alpha_{\mathrm{ST},j}\;\left(j=x,y,z\right)=\cos a_{\mathrm{ST},j}=\frac{{\left(S^{↑}-S^{↓}\right)}_{j}}{\left|(S^{↑}-S^{↓}\right|}=\frac{d_{\mathrm{ST},j}}{D_{\mathrm{ST}}}$$

express the degree of alignment of the components of **d**_CT_ with (**R** ^+^ – **R** ^–^) or of **d**_ST_ with (**S** ^↑^ – **S** ^↓^), where *a*_CT,*j*_ and *a*_ST,*j*_ are the angles between the *j* axis and the (**R** ^+^ – **R** ^–^) or the (**S** ^↑^ – **S** ^↓^) vectors, respectively.

Equations 10 and 13 show that, upon excitation, the magnitude of the change in the dipole moment, ∥**μ**_CT_∥, or the magnitude of the spin density dipole moment, ∥**μ**_ST_∥, resulting from the spin-up and spin-down transfer, **μ**_ST_, is determined by the products of *D*_CT_ and *q*_CT_ or *D*_ST_ and *s*_ST_, respectively. However, their decomposition into contributions from different subdomains is not straightforward for two main reasons. The first reason is that a separate decomposition should be introduced for each **μ**_CT_ and **μ**_ST_ component:

24a $$\mu_{\mathrm{CT},j}\left(Y_{n}\leftarrow Y_{0}\right)=d_{\mathrm{CT},j}\left(Y_{n}\leftarrow Y_{0}\right)\cdot q_{\mathrm{CT}}\left(Y_{n}\leftarrow Y_{0}\right)=\sum_{\Omega }d_{\mathrm{CT},j}^{\Omega }\left(Y_{n}\leftarrow Y_{0}\right)\cdot \sum_{\Omega }q_{\mathrm{CT}}^{+}\left(\Omega ,Y_{n}\leftarrow Y_{0}\right)\equiv \sum_{\Omega }d_{\mathrm{CT},j}^{\Omega }\left(Y_{n}\leftarrow Y_{0}\right)\cdot \sum_{\Omega }q_{\mathrm{CT}}^{-}\left(\Omega ,Y_{n}\leftarrow Y_{0}\right),j=x,y,z$$

24b $$\mu_{\mathrm{ST},j}\left(Y_{n}\leftarrow Y_{0}\right)=d_{\mathrm{ST},j}\left(Y_{n}\leftarrow Y_{0}\right)\cdot s_{\mathrm{ST}}\left(Y_{n}\leftarrow Y_{0}\right)=\sum_{\Omega }d_{\mathrm{ST},j}^{\Omega }\left(Y_{n}\leftarrow Y_{0}\right)\cdot \sum_{\Omega }s_{\mathrm{ST}}^{↑}\left(\Omega ,Y_{n}\leftarrow Y_{0}\right)\equiv \sum_{\Omega }d_{\mathrm{ST},j}^{\Omega }\left(Y_{n}\leftarrow Y_{0}\right)\cdot \sum_{\Omega }s_{\mathrm{ST}}^{↓}\left(\Omega ,Y_{n}\leftarrow Y_{0}\right),j=x,y,z$$

The second reason, and the more challenging issue, is that the products in eqs 35 and 36 involve mixed terms that include pairs of subdomains. While a formal decomposition of each **μ**_CT,*j*_ (or **μ**_ST,*j*_) component into subdomains is possible, for example, by assigning half of each subdomain pair’s contributions to each subdomain, this partitioning remains arbitrary. Therefore, we prefer not to utilize it in this manuscript.

However, the properties of the nonsymmetric square matrix *M*_CT_^*j*,+^ whose diagonal elements represent the single subdomain contributions **d**_CT,*j*_^Ω^·*q*_CT_^+^(Ω) and whose off-diagonal elements represent the mixed terms **d**_CT,*j*_^Ω^·*q*_CT_^+^(Ω′) have been shown to be of significant interest and worth investigating. For instance, each **μ**_CT,*j*_ can be straightforwardly decomposed into an intra-subdomain contribution, **μ**_CT,*j*_^intra,+^, and an inter-subdomains counterpart, **μ**_CT,*j*_^inter,+^, as follows:

25 $$\mu_{\mathrm{CT},j}^{+}\left(Y_{n}\leftarrow Y_{0}\right)=\sum_{i}M_{\mathrm{CT},\mathrm{ii}}^{j,+}+\sum_{i\neq k,k}M_{\mathrm{CT},\mathrm{ik}}^{j,+}=\mu_{\mathrm{CT},j}^{\mathrm{intra},+}\left(Y_{n}\leftarrow Y_{0}\right)+\mu_{\mathrm{CT},j}^{\mathrm{inter},+}\left(Y_{n}\leftarrow Y_{0}\right)$$

The *M*_CT_^*j*,+^ matrix is of dimension *n*_sub_ × *n*_sub_, where *n*_sub_ is the number of subdomains considered. Clearly, an analogous matrix *M*_CT_^*j*,–^ is defined, with diagonal elements corresponding to the single subdomain contributions **d**_CT,*j*_^Ω^·*q*_CT_^–^(Ω) and off-diagonal elements corresponding to the mixed terms **d**_CT,*j*_^Ω^·*q*_CT_^–^(Ω′). Both *M*_CT_^*j*,+^ and *M*_CT_^*j*,–^ matrices have the property that they reproduce the value of ∥**μ**_CT_∥ either from their **μ**_CT,*j*_^+^ or **μ**_CT,*j*_^–^ vector components, or equivalently, by summing all the elements of *M*_CT_^*j*,+^ (eq 37) or *M*_CT_^*j*,–^. However, the corresponding elements of these matrices generally differ from one another.

The decomposition of **μ**_CT,*j*_ provided by eq 37 offers a measure of the degree of interdependency between subdomains in determining the variation of the *j* component of the dipole moment upon excitation. The diagonal elements, *M*_CT,*ii*_^*j*,+^, and the off-diagonal elements, *M*_CT,*ik*_^*j*,+^ (where *M*_CT,*ik*_^*j*,+^ ≠ *M*_CT,*ki*_^*j*,+^ in general), represent the internal contribution of subdomain Ω_*i*_ and the contribution from the subdomain pair Ω_*i*_Ω_*k*_, respectively.

In the case of spin-up and spin-down transfer, a similar approach can be adopted by introducing the asymmetric square matrix *M*_ST_^*j*,↑^, where the diagonal elements correspond to the contributions from individual subdomains, **d**_ST,*j*_^Ω^·*s*_ST_^↑^(Ω), and the off-diagonal elements represent the mixed terms **d**_ST,*j*_^Ω^·*s*_ST_^↑^(Ω′). Each **μ**_ST,*j*_ can, similarly to the charge transfer case, be decomposed into an intra-subdomain contribution, **μ**_ST,*j*_^intra,↑^, and an inter-subdomain counterpart **μ**_ST,*j*_^inter,↑^:

26 $$\mu_{\mathrm{ST},j}^{↑}\left(Y_{n}\leftarrow Y_{0}\right)=\sum_{i}M_{\mathrm{ST},\mathrm{ii}}^{j,↑}+\sum_{i\neq k,k}M_{\mathrm{ST},\mathrm{ik}}^{j,↑}=\mu_{\mathrm{ST},j}^{\mathrm{intra},↑}\left(Y_{n}\leftarrow Y_{0}\right)+\mu_{\mathrm{ST},j}^{\mathrm{inter},↑}\left(Y_{n}\leftarrow Y_{0}\right)$$

Similarly to the charge transfer case, a matrix *M*_ST_^*j*,↓^ is also defined, with diagonal elements corresponding to the contributions from individual subdomains **d**_ST,*j*_^Ω^·*s*_ST_^↓^(Ω) and off-diagonal elements representing the mixed terms **d**_ST,*j*_^Ω^·*s*_ST_^↓^(Ω′). For transitions involving states with the same quantum spin *S*, both *M*_ST_^*j*,↑^ and *M*_ST_^*j*,↓^ matrices have the property that they reproduce the ∥**μ**_ST_∥ value from either their **μ**_ST,*j*_^↑^ or **μ**_ST,*j*_^↓^ vector components, or equivalently, by summing up all the elements of either the *M*_ST_^*j*,↑^ (eq 38) or *M*_ST_^*j*,↓^ matrices. As for the charge transfer case, it is anticipated that the corresponding matrix elements of these matrices generally differ from one another. Hence, the intra-subdomain contributions (**μ**_ST,*j*_^intra,↑^ and **μ**_ST,*j*_^intra,↓^) and the inter-subdomain contributions (**μ**_ST,*j*_^inter,↑^ and **μ**_ST,*j*_^inter,↓^) will generally differ as well. The **μ**_ST,*j*_ decomposition (eq 38) measures the extent of subdomain interdependency in determining the *j* component of the spin density dipole moment, resulting from the spin-up and spin-down transfer. The diagonal elements, *M*_ST,*ii*_^*j*,↑^, and the off- diagonal elements, *M*_ST,*ik*_^*j*,↑^, with *M*_ST,*ik*_^*j*,↑^ ≠ *M*_ST,*ki*_^*j*,↑^ in general, represent, respectively, the internal contribution of subdomain Ω_*i*_ and the pair contributions between subdomains Ω_*i*_ and Ω_*k*_ to the spin density dipole moment. Clearly, a similar interpretation holds for the corresponding elements of *M*_ST_^*j*,↓^.

A much simpler subdomain decomposition of ∥**μ**_CT_∥ (or ∥**μ**_ST_∥) can be achieved by considering only the dependence of∥**μ**_CT_∥ (or ∥**μ**_ST_∥) on *q*_CT_ (or *s*_ST_). Under this assumption, ∥**μ**_CT_∥ and ∥**μ**_ST_∥ are simply given by:

27a $$||\mu_{\mathrm{CT}}||=D_{\mathrm{CT}}\cdot q_{\mathrm{CT}}=D_{\mathrm{CT}}\cdot \sum_{\Omega }q_{\mathrm{CT}}^{+}\left(\Omega \right)\equiv D_{\mathrm{CT}}\cdot \sum_{\Omega }q_{\mathrm{CT}}^{-}\left(\Omega \right)$$

27b $$||\mu_{\mathrm{CT}}||=\sum_{\Omega }\mu_{\mathrm{CT}}^{+}\left(\Omega \right)\equiv \sum_{\Omega }\mu_{\mathrm{CT}}^{-}\left(\Omega \right)$$

27c $$||\mu_{\mathrm{ST}}||=D_{\mathrm{ST}}\cdot s_{\mathrm{ST}}=D_{\mathrm{ST}}\cdot \sum_{\Omega }s_{\mathrm{ST}}^{↑}\left(\Omega \right)\equiv D_{\mathrm{ST}}\cdot \sum_{\Omega }s_{\mathrm{ST}}^{↓}\left(\Omega \right)$$

27d $$||\mu_{\mathrm{ST}}||=\sum_{\Omega }\mu_{\mathrm{ST}}^{↑}\left(\Omega \right)\equiv \sum_{\Omega }\mu_{\mathrm{ST}}^{↓}\left(\Omega \right)$$

Equations 39–42 allow us to obtain a single value for ∥**μ**_CT_∥ (or ∥**μ**_ST_∥), but expressed in terms of two different subdomain decompositions, one based on *q*_CT_^+^(Ω) (or *s*_ST_^↑^(Ω)) and the other based on *q*_CT_^–^(Ω) (or *s*_ST_^↓^(Ω)). These alternative subdomain decompositions of ∥**μ**_CT_∥ (or ∥**μ**_ST_∥) provide insight into the roles of *q*_CT_^+^(Ω) and *q*_CT_^–^(Ω) (or of *s*_ST_^↑^(Ω) and *s*_ST_^↓^(Ω)) in determining ∥**μ**_CT_∥ (or ∥**μ**_ST_∥). Both decompositions have chemical significance, and this is true for both ∥**μ**_CT_∥ and ∥**μ**_ST_∥.

### Implementing the Atomic Group Decomposition of Charge and Spin Transfer Global Indexes

As previously stated, eqs 14–31 and 35–39 remain valid regardless of whether a fuzzy boundary or a disjoint exhaustive space partitioning scheme is used. However, adopting the Quantum Theory of Atoms in Molecules (QTAIM)^25^ zero-flux condition to define the subdomains Ω,

$$\nabla \varrho \left(r_{s}\right)\cdot n\left(r_{s}\right)=0\;\forall \;r_{s}\in S$$

where **r**_***s***_ is any point on the subdomain boundary surface *S* and ***n*** (**r**_***s***_) is the normal vector at **r**_***s***_, allows for the rigorous association of the subdomain contributions to charge transfer (CT) and spin transfer (ST) indexes with atoms or group of atoms as defined by quantum mechanics.^25^

Equations 14–31 and 35–39 pertain to two-state quantities. Since the QTAIM space partitioning—like any partitioning that is not purely geometrical—is state-dependent, it becomes necessary to choose one of the two electronic states involved as the reference for atomic and group partitioning in these equations. To ensure consistency across a series of excited states for a given molecule, we have always adopted the space partitioning corresponding to the ground state (GS) as the common reference. It is important to note that, similar to the LBAC model, only vertical excitations can be considered in the subdomain formulation of this model. Therefore, any change in the subdomain boundaries upon excitation is expected to be significantly dampened compared to what would occur in adiabatic electronic transitions. In other words, whether the atomic boundaries are derived from the ground or the excited state should not significantly alter the distribution of global CT indexes into atomic or group contributions.

Building on the premises outlined above, in our previous work, we developed a code called DOCTRINE^24^ (atomic group *Decomposition Of the Charge TRansfer INdExes*), which, as a first step, computes the contributions of all QTAIM atomic basins to the CT indexes. The updated version presented in this paper, DOCTRINE_SPIN,^35^ extends this capability to the ST indexes as well.

At this stage of the calculation, the domain Ω in eqs 14–31 and 35–39 corresponds to any atomic basin of the molecule. Like its predecessor, DOCTRINE_SPIN uses wavefunction files in .wfn format—generated with the *ab initio* GAUSSIAN-16 code^36^—for both the ground and the target excited state *n* of the molecule under investigation. Additionally, it requires a .sur file containing the surface boundary information of all QTAIM atomic basins Ω in the ground state. This file is produced in a preliminary step by a PROMEGA calculation (part of Bader’s AIMPAC95 package^37^), which determines the boundaries of all atomic basins in the ground state molecule. If desired, integral properties of the QTAIM basins can also be computed at this stage to explore potential correlations with the CT and ST indexes and their decomposition into atomic group contributions.^24,26^ In such cases, analogous calculations must be performed for the excited state to enable analysis of the changes in integral properties upon electronic excitation.

As in the original DOCTRINE code, DOCTRINE_SPIN evaluates ∥**μ**_CT_∥(*Y*_*n*_ ← *Y*_0_) using eq 10 (or alternatively via eq 35 or eq 39–40). The accuracy of the numerical integration can be assessed by comparing the reconstructed value of ∥**μ**_CT_∥(*Y*_*n*_ ← *Y*_0_) obtained from the sum of contributions over all atomic basins Ω with the value derived from the dipole moment vector components of the *Y*_*n*_ and *Y*_0_ states as calculated by the GAUSSIAN 16 code. Since this is performed for all QTAIM atoms in spherical coordinates and on an atomic-centered grid, the resulting accuracy is noteworthy, provided a suitable number of angular and radial points is used in the Gaussian quadrature integration procedure (see the Computational Details section). By contrast, an analogous consistency check cannot be performed for the norm of the spin-density dipole moment arising from spin-up and spin-down transfer, as neither the spin dipole nor its individual components are available from GAUSSIAN.

Further checks of the integration accuracy—this time involving also spin density–dependent quantities—can be performed by comparing values that should, in principle, be equivalent. These include ∥**μ**_CT_∥ and ∥**μ**_ST_∥, calculated using either *q*_CT_^+^ (Ω) or *q*_CT_^–^ (Ω), and *s*_ST_^↑^ (Ω) or *s*_ST_^↓^ (Ω), respectively (see eqs 39–40,41–42). Similarly, *q*_CT_ and *s*_ST_ values can be computed using either ρ^+^ (***r***) or ρ^–^ (***r***), and *s* ^+^(***r***) or *s* ^–^(***r***), respectively (see eqs 14 and 15), allowing for internal consistency checks within the different formulations.

Once the QTAIM atomic basin contributions to the CT and ST indexes have been determined, DOCTRINE_SPIN combines and aggregates them into the user-defined *nsub* atomic group contributions. Each group corresponds to a disjoint subset of atoms, selected to represent chemically or functionally meaningful subdomains of the molecule. Naturally, the union of all *nsub* groups must account for the entire atomic composition of the system under investigation.

## Application of the Developed Method to the TMTQ System

As already stated, for the initial application of our spin-polarized model extension, we have selected a π-conjugated compound (TMTQ), which consists of a central 1,6-methano[10]­annulene (M10A) core and two 5-dicyanomethyl-thiophene (DT) units at the periphery in an exo configuration.^33,34^ It has been proposed^33^ that in TMTQ, the DT units may act as electron acceptors (A) due to the strong electron-withdrawing character of the dicyanomethylene groups. Conversely, the nonaromatic annulene core of TMTQ, with its highly distorted and quinoidal structure in the singlet ground state, is considered to facilitate electron donation, thereby enabling M10A to function as an electron donor (D). This A–D–A architecture of TMTQ is regarded as an ideal configuration to promote excited state intramolecular charge transfer (iCT) processes.^33^ In this symmetric A–D–A geometry, the iCT process is expected to involve the transfer of two π-electrons from the M10A core to the DT units. According to Baird’s rule^38–40^ —which has been shown to apply to excited states of arbitrary spin^41,42^ —this electron redistribution could induce aromatization of the core annulene by converting its 10 π-electrons in the ground state into 8 π-electrons in the CT state. An increase in aromatic character has indeed been observed in both the S_1_ and T_1_ states relative to the ground state (S_0_), but the proposed loss of two π-electrons from M10A to the DT units in these more aromatic excited states has been subject to critical examination.^34^ Specifically, analyses of charge and spin density distributions have demonstrated that the T_1_ state may be described as a Hückel–Baird hybrid. This hybrid nature allows for a tunable balance between a closed-shell Hückel aromaticity (4n+2 π-electrons, with the annulenic core retaining 10 π-electrons) and an open-shell Baird aromaticity (4n π-electrons, with the annulenic core doubly positively charged and retaining only 8 π-electrons). However, because the Hückel-aromatic character predominates in the triplet state^34^—with the Baird aromatic contribution not exceeding 12%—TMTQ is more accurately described as a Hückel-aromatic compound influenced by Baird aromaticity.^34^ The small singlet–triplet energy gap has, in fact, been attributed^34^ to the Hückel aromaticity of the M10A core.

A more detailed analysis of the extent of charge transfer from the putative donor (D) to the two acceptor (A) moieties of TMTQ upon excitation will be presented in the following sections. For the purposes of the present study, it is important to note: (*i*) that TMTQ is capable of undergoing substantial intramolecular charge transfer (iCT) processes, which involve changes in the molecule’s electronic conjugation and aromatic character; and (*ii*) that the magnitude and nature of these changes may vary depending on whether the process involves only charge transfer or both charge and spin transfer.

## Computational Details

Quantum chemical calculations were performed using Density Functional Theory (DFT) and Time-Dependent DFT (TD-DFT) as implemented in the Gaussian 16 software package^36^. All DFT and TD-DFT calculations employed the long-range corrected (LRC), Coulomb-attenuated B3LYP exchange-correlation functional (CAM-B3LYP)^43^ combined with the cc-pVDZ basis set. Solvent effects were modeled implicitly using the SMD solvation model^44^ with nitromethane as the solvent. The DFT-optimized geometry of TMTQ-1 in its singlet ground state (S_0_) was obtained using tight convergence criteria. The nature of the optimized structure as a local energy minimum was confirmed by harmonic vibrational analysis (no imaginary frequencies). TD-DFT calculations were then used to determine the first five vertical excitations of TMTQ-1 (Franck–Condon states). Similarly, we also investigated the structural and electronic properties of the biradical TMTQ-3 (total spin 1) in its ground state (T_1_), as well as its first five vertical triplet–triplet excitations (T_1_ → T_*n*_). In addition, to evaluate the potential impact of variations in the computational model—particularly the role of electron correlation (dispersion) energy, basis set effects, and the influence of the surrounding solvent on the solute’s properties—on the relative weights of nearly degenerate mesomeric structures with distinct electron delocalization patterns, we carried out additional DFT calculations for both the singlet ground (S_0_) and biradical (T_1_) states of TMTQ in the gas phase. These calculations were carried out at the B3LYP-D3­(BJ)/6-311G­(d,p) and CAM-B3LYP-D3­(BJ)/cc-pVDZ levels, respectively (see next section). The calculated electronic energies (*E*°), zero-point energy–corrected electronic energies (*E*° + ZPE), internal energies, enthalpy, and Gibbs free energies at 298 K, along with geometrical coordinates are reported in Tables S01–S03.

The .wfn files for the singlet and triplet ground states (S_0_ and T_1_), as well as for the (5+5) excited states considered in this study, were obtained from corresponding GAUSSIAN-16 calculations. Atomic boundaries for the S_0_ and T_1_ ground states were determined using the PROMEGA code, typically employing 6144 angular grid points (96 for φ and 64 for θ). The evaluation of atomic contributions to the global charge transfer (CT) indexes by the DOCTRINE_SPIN code was performed using 200 radial points in the Gaussian quadrature integration outside the so-called β sphere, along with the same angular grid used in the PROMEGA boundary determination. The evaluation of atomic contributions to the global CT indexes is approximately 2 orders of magnitude faster than the atomic boundary determination and is therefore computationally inexpensive (typically requiring only 5–10 min for a system like TMTQ on a medium-sized workstation cluster). Importantly, the most CPU-intensive step—atomic boundary determination—needs to be performed only for the ground states (singlet and triplet, S_0_ and T_1_) of a given system, regardless of the number of excited states investigated.

The following four moieties of TMTQ-1 (or TMTQ-3) were considered in our CT and ST analysis using DOCTRINE_SPIN: (i) the central 1,6-methano[10]­annulene (M10A) core, (ii) the two thiophene units (THIO1 and THIO2), and (iii) the two peripheral dicyanomethyl groups (DCN1 and DCN2) (Figure 1). It is worth noting that, although the formal molecular symmetry of the singlet and triplet ground states (S_0_ and T_1_) is C_2_, optical excitation may induce electron distributions that differentiate the two thiophene units and the two dicyanomethyl groups from each other.

**Figure 1:**
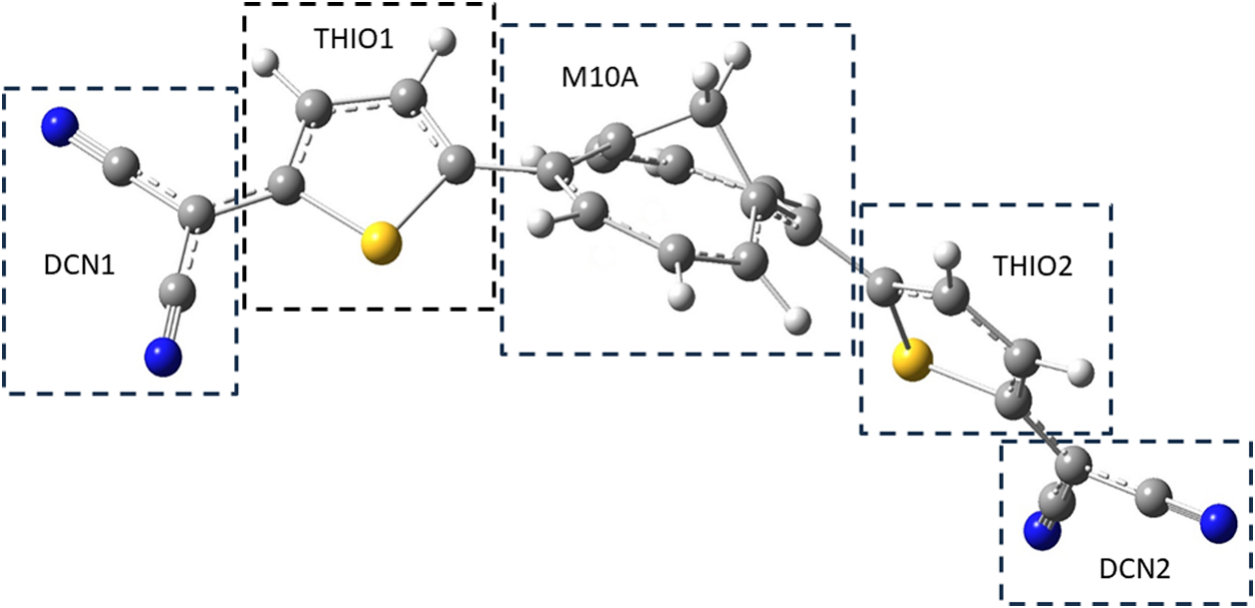
Molecular moieties and their corresponding labels used in the decomposition of the global charge and spin transfer indexes of TMTQ into contributions from atomic groups. The molecular geometry shown corresponds to the triplet ground state.

## Results and Discussion

### Accuracy of Global and Atomic Group Charge and Spin Transfer Indexes

Table S1 presents, for all investigated excitations, the values of several quantities used to assess the accuracy (as discussed earlier) of the computed global and atomic group charge and spin transfer indexes. The quantity Δ∥**μ**_CT_∥ = ∥**μ**_CT_∥_DOCTRINE_ – ∥**μ**_CT_∥_Gaussian 16_ (reported in column 2) represents the difference between the ∥**μ**_CT_∥ value obtained from the sum of contributions of individual atomic or atomic group subdomains, as evaluated by the DOCTRINE_SPIN code, and the ∥**μ**_CT_∥ value derived from the dipole moment components of the two involved electronic states, as computed using Gaussian-16. The largest Δ∥**μ**_CT_∥ value is negligibly small (−0.0025 D) (D = Debye), especially when compared to a ∥**μ**_CT_∥ value as large as 0.898 D for the S_4_ ← S_0_ excitation.

Table S1 also reports the amounts of transferred charge (*q*_CT_) and of spin-up and spin-down transferred electron charges (*s*_ST_^↑^ and *s*_ST_^↓^) calculated upon excitation by integrating either ρ^+^(***r***) or ρ^–^(***r***), and either *s* ^+^(***r***) or *s* ^–^(***r***), over all atomic or atomic groups subdomains of the TMTQ molecule. The discrepancies between *q*_CT_^+^ and *q*_CT_^–^, and between *s*_ST_^↑^ and *s*_ST_^↓^, are again negligibly small—at most 3 × 10^–4^ e^–^ and 8 × 10^–5^ e^–^, respectively, in the worst cases. In most instances, these differences are 1 to 2 orders of magnitude smaller. Overall, the data reported in Table S1 demonstrate the high integration accuracy of the charge and spin transfer indexes computed using the DOCTRINE_SPIN code.

### Changes in TMTQ Subdomains Net Charges, Spin Populations, and TMTQ Electron Delocalization/Aromaticity Indices upon Excitation

Table 1 presents data, in e^–^, on changes in the net charges, Δ*q*_Ω_, and spin populations, Δ*s*_Ω_, of molecular subdomains of the TMTQ molecule in both singlet and triplet states, upon excitation. These values are computed as the difference between the subdomain Bader’s net charges and spin populations in the excited and reference states. The analysis is conducted for the molecular subdomains Ω denoted as DCN = DCN1∪DCN2, THIO = THIO1∪THIO2, and M10A. Note that positive values of Δ*q*_Ω_ and Δ*s*_Ω_ indicate a decrease in the number of electrons and an increase in their α-spin component within the subdomain upon excitation. This contrasts with the convention adopted for charge transfer (Δ*q*_CT_(Ω)), where a positive value of Δ*q*_CT_(Ω) signifies an increase in the electron population of the subdomain, upon excitation, rather than a decrease. For the reference states S_0_ and T_1_, the net charge *q*_Ω_ and spin population *s*_Ω_ in each subdomain (M10A, THIO, and DCN) are reported, along with their respective differences, Δ*q*_Ω_ and Δ*s*_Ω_, calculated as T_1_–S_0_.

**Table 1:** TMTQ in Nitromethane (DFT and TD-DFT at CAM-B3LYP/cc-pVDZ Level)^a^

Excitation	Δ*q*_M10A_	Δ*s*_M10A_	Δ*q*_THIO_	Δ*s*_THIO_	Δ*q*_DCN_	Δ*s*_DCN_	Δ_HOMA_ ^b^	Δ_FLU_ ^b^	Δstd_R_ ^b^	Δstd_DI_ ^b^	Δ*R*_av_ ^b^	ΔDI_av_ ^b^
S_1_ ← S_0_	0.062	-	–0.048	-	–0.018	-	-	–0.017	-	–0.076	-	0.007
T_2_ ← T_1_	*0.148*	*0.188*	*–0.063*	*–0.061*	*–0.081*	*–0.127*	*-*	*0.002*	*-*	*0.004*	*-*	*0.011*
S_2_ ← S_0_	0.037	-	–0.042		0.006	-	-	–0.005	-	–0.019	-	0.010
T_3_ ← T_1_	*0.208*	*0.307*	*–0.067*	*–0.008*	*–0.148*	*–0.259*	*-*	*–0.005*	*-*	*–0.007*	*-*	*0.045*
S_3_ ← S_0_	-0.002	-	–0.037	-	0.038	-	-	–0.014	-	–0.065	-	0.003
T_4_ ← T_1_	*0.130*	*0.201*	*–0.026*	*–0.052*	*–0.101*	*–0.149*	*-*	*–0.001*	*-*	*–0.003*	*-*	*0.009*
S_4_ ← S_0_	0.349	-	–0.181	-	–0.168	-	-	–0.019	-	–0.110	-	–0.017
T_5_ ← T_1_	*0.432*	*0.628*	*–0.147*	*–0.215*	*–0.282*	*–0.413*	*-*	*–0.003*	*-*	*0.000*	*-*	*0.025*
S_5_ ← S_0_	0.059	-	–0.047	-	–0.011	-	-	–0.009	-	–0.042	-	–0.007
T_6_ ← T_1_	*-0.008*	*-0.049*	*0.201*	*0.501*	*–0.192*	*–0.452*	*-*	*–0.006*	*-*	*–0.012*	*-*	*0.052*
T_1_ ← S_0_	-0.171	0.227	–0.005	0.769	0.169	1.003	0.815	–0.026	-0.039	–0.174	–0.012	–0.029

State	*q*_M10A_	*s*_M10A_	*q*_THIO_	*s*_THIO_	*q*_DCN_	*s*_DCN_	HOMA^b^	FLU^b^	std_R_ ^b^	std_DI_ ^b^	*R*_av_ ^b^	DI_av_ ^b^
S_0_	0.475	-	0.390	-	–0.853	-	0.039	0.037	0.052	0.246	1.420	1.287
T_1_	0.304	0.227	0.385	0.769	–0.684	1.003	0.854	0.011	0.013	0.072	1.408	1.258

a Bader’s Net Charge (Δ*q*_Ω_) and spin population (Δ*s*_Ω_) changes in the singlet and triplet TMTQ molecule subdomains Ω upon excitation, along with the corresponding changes in the values of descriptors related to electron delocalization in the annulenic ring (M10A). Net charges, spin populations, and their changes upon excitation are reported, in e^–^, for the molecular subdomains: DCN = DCN1∪DCN2, THIO = THIO1∪THIO2, and M10A. For the reference S_0_ and T_1_ states, the net charges and spin populations, in e^–^, of the M10A, THIO, and DCN subdomains are reported, together with geometrical and electronic descriptors related to electron delocalization in the M10A annulenic ring. All numeric data for excitations between triplet states in *italic*.

b HOMA and FLU are geometric and electronic descriptors of electronic delocalization (see text). *R*_av_, in Å, is the average C–C bond distance, and DI_av_ is the average delocalization index, while std_R_, in Å, and std_DI_ are their associated standard deviations. The changes in all these quantities *Z* upon excitation (final – initial state) are denoted as Δ*Z* (last 6 columns for state excitation changes). For vertical excitations, the changes in the geometrical descriptors are zero by definition and are not reported. Geometric and electronic descriptors are evaluated using only data of the annulenic 10-CMR (10-Carbon Membered Ring) bonds, *i.e.*, the data for bonds including the apical C atom are not included.

Net charges, spin populations, and their changes are analyzed in relation to geometric and electronic descriptors of π-electron delocalization within the 10-membered annulenic carbon ring (10-CMR), also listed in Table 1. Geometry descriptors include the Harmonic Oscillator Model of Aromaticity (HOMA),^45^ the average C–C bond length *R*_av_, and the standard deviation of these bond lengths, std_R_, within the ring. HOMA quantifies π-electron delocalization in cyclic π-conjugated molecules based on bond lengths: a value of 1 indicates perfect aromaticity, while values of 0 or even negative indicate nonaromatic character. Electronic descriptors of delocalization include the aromatic fluctuation index FLU^46^, the average delocalization index,^47^ DI_av_, and its standard deviation, std_DI_, all referring to the π-electron delocalization across the C–C bonds in the annulenic ring. The delocalization index,^47^ DI (I,J), measures the number of electrons shared between atomic basins I and J. Its average value reflects the mean C–C bond order in the ring, while the standard deviation indicates the degree of deviation from a uniform delocalization. This deviation is conceptually similar to FLU, which quantifies the variance of C–C bond delocalization indices relative to their uniform value in benzene. FLU is zero for benzene and increases with decreasing delocalization uniformity. Changes in these electronic descriptors upon excitation, ΔZ, where Z = FLU, DI_av_, and std_DI_ are also reported in Table 1. Since vertical excitations are considered, geometric descriptors of electron delocalization (HOMA, *R*_av_, and std_R_) remain unchanged upon excitation.

Data in Table 1 show that the net charge of the M10A subdomain in the ground triplet state (*q*_M10A_ = 0.304 e^–^) is significantly lower than the value of two, which would be expected if the iCT process (from S_0_ to T_1_) involved the transfer of two π-electrons from the M10A core to the DT units. Furthermore, the spin population on the M10A subdomain is only 0.227 e^–^, again far below the value of two expected for a fully Baird-aromatic ^3^M10A^2+^species. The fact that only a minor portion of the spin density is localized on the M10A moiety is consistent with its low net charge. Based on charge and spin density considerations, the degree of Baird aromaticity is limited by the spin distribution and amounts to at most 11,3%, [(0.227/2)*100]. These findings are in qualitative agreement with ref 34, which describes the TMTQ ground state triplet as a closed-shell Hückel-aromatic compound (4n+2 π-electrons), where the annulenic core retains 10-π-electrons and is only slightly influenced by Baird aromaticity. Interestingly, the net charge of the M10A subdomain in the ground singlet state is even higher (*q*_M10A_ = 0.475 e^–^) than in the T_1_ state (*q*_M10A_ = 0.304 e^–^). However, both geometric and electronic descriptors of π-electron delocalization and aromaticity suggest that the annulenic ring is nearly aromatic in the T_1_ state and almost nonaromatic in the S_0_ state. Taken together, the results (Figure 2) support a description of singlet TMTQ as being primarily represented by the quinoidal/covalent resonance structure **a**, with minor contributions from the Baird-aromatic (8-π electrons)/polar structure **c**. In contrast, the triplet TMTQ is best described by the diradical/ Hückel-aromatic (10-π electrons) structure **b**, with a slight contribution from the Baird aromatic (8-π electrons)/polar structure **c**.

**Figure 2:**
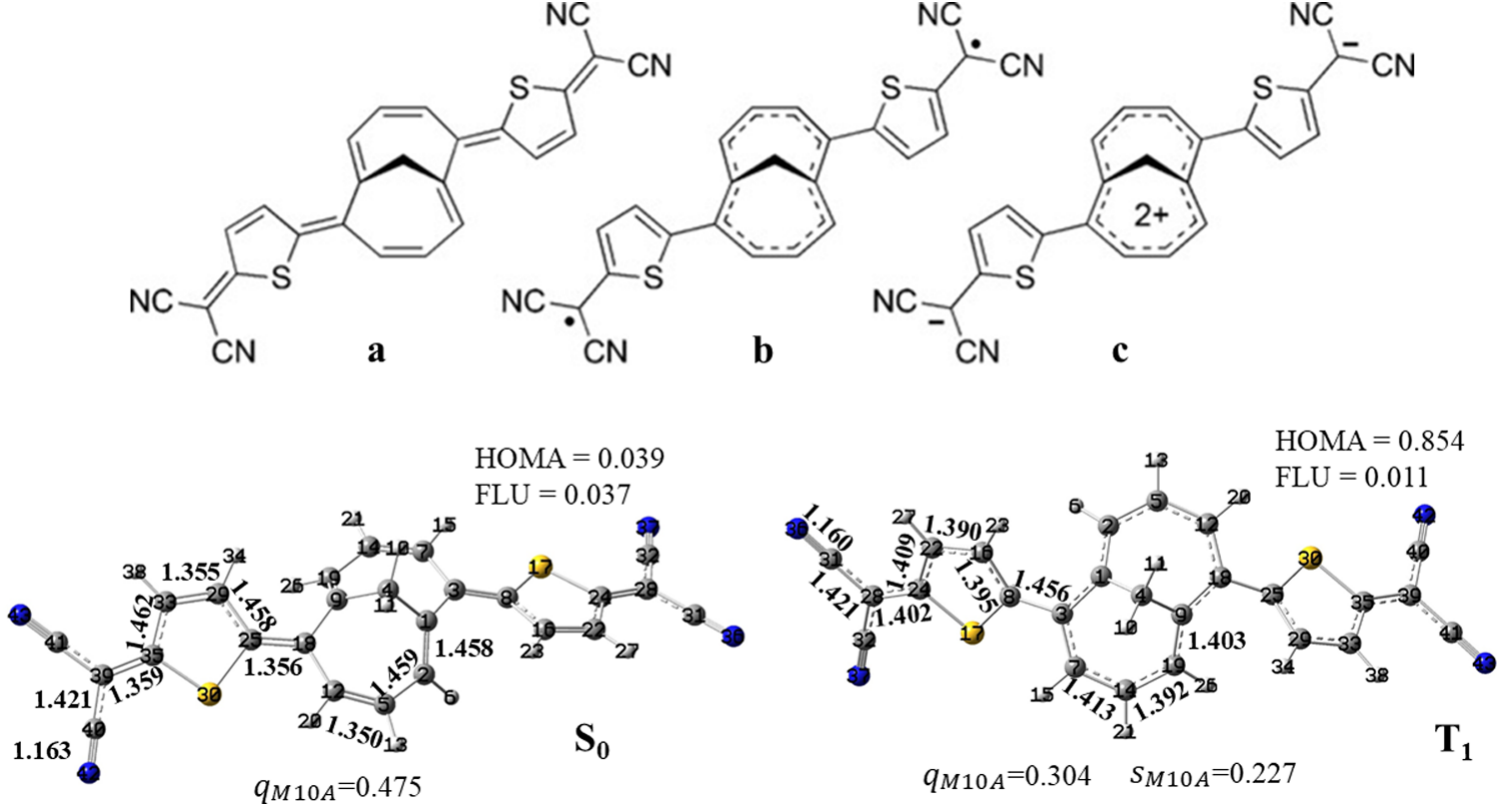
TMTQ molecule in nitromethane (DFT and TD-DFT at CAM-B3LYP/cc-pVDZ level). *Top*: Quinoidal/Covalent (**a**), diradical/Hückel-aromatic (10-π electrons) (**b**), and polar (/Baird aromatic (8-π electrons)) (**c**) mesomeric structures of importance for TMTQ molecule. Adapted from Figure 1 of ref 34, Copyright 2016 Wiley-VCH Verlag 15530 GmbH&Co. KGaA; *Bottom*: C–C and C-N bond distances (Å) for the quinoidal S_0_ and for the T_1_ state. The latter state is best classified as a Hückel-aromatic compound influenced by Baird aromaticity.

#### Effects of Changes in the Computational Approach

The sensitivity of the above conclusions to model and basis set effects, as well as to solvent effects, is examined in Tables S2–S4. These tables report the same quantities listed in Table 1 but refer to DFT and TD-DFT computations carried out *in vacuo* at different levels of theory: B3LYP-D3­(BJ)/6-311G­(d,p) (Table S2), CAM-B3LYP-D3­(BJ)/cc-pVDZ (Table S3), and CAM-B3LYP/cc-pVDZ (Table S4). Table S4 differs from Table 1 only in the dielectric environment employed (*in vacuo vs.* nitromethane, respectively). Table S3 introduces the additional effect of dispersion corrections through Grimme’s D3 model with Becke–Johnson damping [DFT-D3­(BJ), Empirical Dispersion=GD3BJ],^48^ relative to Table S4. Table S2, in addition to being *in vacuo*, employs a different basis set [6-311G­(d,p)][Bibr ref49] and functional (B3LYP).^50,51^ The computational level used in Table S2 corresponds to that adopted in ref 52, and is similar to the levels used for TMTQ in [refs 33,34 ]—namely, B3LYP/6-31G­(d,p) [refs 33,34 ] and B3LYP/6-311+G­(d,p) [ref 34].

The results in Tables S2–S4 for both singlet and triplet ground states are qualitatively consistent with those in Table 1. They confirm that the net charge on the M10A subdomain in the triplet ground state is significantly lower than 2, and even lower than in the singlet ground state (S_0_), regardless of changes in the DFT functional, basis set, or inclusion of dispersion corrections. The latter, in particular, have only a minor influence. In all cases—except for the B3LYP-D3­(BJ)/6-311G­(d,p) model (Table S2)—the degree of Baird aromaticity is constrained by the spin distribution and amounts to 12.9, 8.8, and 8.8% for the models in Tables S2, S3, and S4, respectively, compared to 11.3% reported in Table 1. All models consistently show that the annulene ring is nearly aromatic in the T_1_ state and nearly nonaromatic in the S_0_ state, as supported by both geometric (HOMA, std_R_) and electronic (FLU, std_DI_) descriptors of π-electron delocalization and aromaticity. As expected, *in vacuo* calculations yield significantly lower charge and spin separation among the TMTQ moieties.

When comparing net charges and spin population changes upon excitation, dispersion effects remain negligible (*cf.* Tables S3 *vs.* S4), whereas the solvent medium can have a significant impact (*cf.* Table 1 *vs.* Tables S3–S4). Similarly, the choice of basis set and DFT functional can also substantially affect the results (*cf.* Table S2 *vs.* Tables S3–S4).

### Global Charge Transfer (CT) and Spin Transfer (ST) Indexes and Their Atomic Groups Decomposition

Table 2 presents the global charge transfer (CT) and spin transfer (ST) indexes, along with atomic group decompositions of the transferred charge *q*_CT_ and spin *s*_ST_, for the first five singlet and triplet vertical excited states of TMTQ in nitromethane. Results in Table 2 and in all following tables and figures refer to the DFT and TD-DFT CAM-B3LYP/cc-pVDZ computational level. The global CT and ST indexes shown in Table 2 include the values of *q*_CT_ and *s*_ST_, the magnitude of the charge- and spin-transfer dipole moments between the ground and excited states, and the excitation lengths *D*_CT_ and *D*_ST_ for charge and spin transfer, respectively. The decompositions of *q*_CT_ and *s*_ST_ are presented in terms of either the contributions *q*_CT_^+^(Ω) or *q*_CT_^–^(Ω), and, analogously, of either *s*_ST_^↑^(Ω) or *s*_ST_^↓^(Ω) for spin, for each subdomain Ω. The atomic group decomposition is reported for the molecular subdomains defined as DCN = DCN1∪DCN2, THIO = THIO1∪THIO2, and M10A. Figures 3 and 4 analyze four excitations in more detail, namely the fourth singlet and triplet (Figure 3) and the third singlet and triplet (Figure 4) vertical excited states. In the left panels of these figures, the locations of the positive (or spin-up) and negative (or spin down) centroids of the transferred charge or spin upon excitation are shown as green and red small electronic spheres, respectively, while in the right panels the atomic group decomposition of *q*_CT_ and *s*_ST_ is displayed, for each excitation, for the whole set of subdomains shown in Figure 1, thereby enabling us to distinguish the excitations that break or preserve the molecular symmetry in terms of charge and spin transfer. The excited states shown in Figure 3 have been selected because of their largest dipole moment change, excitation lengths, and both charge and spin transfers among all studied excitations, while those displayed in Figure 4 for the notable asymmetry in the contributions of the two thiophene and two dicyano groups induced by the excitation.

**Table 2:** Global Charge Transfer (CT) and Spin Transfer (ST) Indexes, along with Atomic Group Decompositions of the Transferred Charge *q*_CT_ and Spin *s*_ST_, for the First Five Singlet and Triplet Vertical Excited States of TMTQ in Nitromethane (Computed at the DFT and TD-DFT CAM-B3LYP/cc-pVDZ Levels)^a^

				*q*_CT_^+^(Ω), *s*_ST_^↑^(Ω)			*q*_CT_^–^(Ω), *s*_ST_^↓^(Ω)				Δ*q*_CT_(Ω), Δ*s*_ST_(Ω)		
State	|μ_CT_|, (|μ_ST_|)	*D*_CT_, (*D*_ST_)	*q*_CT_, (*s*_ST_)	DCN	THIO	M10A	DCN	THIO	M10A	*A,B* ^b^	DCN	THIO	M10A
**Singlet states values**													
S_1_ ← S_0_	0.050	0.021	0.496	0.043	0.220	**0.234**	0.036	0.172	**0.287**	0.053	0.006	0.047	**–0.053**
S_2_ ← S_0_	0.182	0.099	0.384	0.045	**0.234**	0.106	0.068	**0.184**	0.132	0.050	–0.024	**0.050**	–0.026
S_3_ ← S_0_	0.349	0.151	0.482	0.036	**0.244**	0.202	0.092	**0.200**	0.191	0.055	**–0.056**	0.044	0.011
S_4_ ← S_0_	0.896	0.235	0.793	0.202	**0.303**	0.287	0.030	0.100	**0.662**	0.375	0.171	0.205	**–0.375**
S_5_ ← S_0_	0.094	0.043	0.450	0.060	**0.234**	0.156	0.058	0.167	**0.224**	0.069	0.002	0.066	**–0.069**
**Triplet states: charge (** ***and spin*** **) values**													
T_2_ ← T_1_	1.250	0.520	0.500	0.112	0.163	**0.226**	0.022	0.084	**0.394**	0.168	0.090	0.079	**–0.168**
	*1.225*	*0.268*	*0.950*	*0.410*	*0.366*	***0.542***	*0.168*	***0.427***	*0.355*	*0.188*	*–0.126*	–0.061	***0.188***
T_3_ ← T_1_	2.269	0.855	0.553	0.216	**0.269**	0.066	0.056	0.193	**0.303**	0.237	0.160	0.076	**-0.237**
	*2.947*	*0.574*	*1.070*	*0.100*	***0.584***	*0.385*	*0.400*	***0.591***	*0.078*	*0.307*	*–0.299*	*–0.008*	***0.307***
T_4_ ← T_1_	1.768	0.917	0.401	**0.145**	0.113	0.143	0.033	0.091	**0.279**	0.135	0.113	0.022	**–0.135**
	*2.025*	*0.639*	*0.661*	*0.047*	*0.291*	***0.324***	*0.195*	***0.343***	*0.123*	*0.201*	*–0.149*	*–0.052*	***0.201***
T_5_ ← T_1_	4.759	1.192	0.831	**0.364**	0.274	0.193	0.054	0.121	**0.655**	0.463	0.310	0.154	**–0.463**
	*6.386*	*1.044*	*1.274*	*0.111*	*0.168*	***0.995***	***0.524***	*0.383*	*0.366*	*0.629*	*–0.413*	*–0.215*	***0.629***
T_6_ ← T_1_	1.516	0.666	0.474	**0.277**	0.146	0.051	0.048	**0.385**	0.041	0.240	0.227	**–0.240**	0.010
	*1.288*	*0.250*	*1.071*	*0.131*	***0.834***	*0.105*	***0.583***	*0.333*	*0.153*	*0.500*	*–0.452*	***0.501***	*–0.049*

a *All numeric data related to spin transfer are shown in italic.* The global CT and ST indexes include the values of *q*_CT_ and *s*_ST_ (in e^–^), the magnitude of the charge- and spin-transfer dipole moments (in Debye, D) between the ground and excited states, and the excitation lengths *D*_CT_ and *D*_ST_ (in Å) for charge and spin transfer, respectively. The decompositions of *q*_CT_ and *s*_ST_ are in terms of the contributions *q*_CT_^+^(Ω) and *q*_CT_^–^(Ω), and analogously *s*_ST_^↑^(Ω) and *s*_ST_^↓^(Ω) for spin. The net charge and spin changes, Δ*q*_CT_(Ω) and Δ*s*_ST_(Ω), are given by the difference between the corresponding positive (or spin up) and negative (or spin down) components. For each excitation, the subdomain contributions with the largest absolute values are highlighted in bold. The values of *A* and *B*, obtained as the sum of only the positive Δ*q*_CT_(Ω) or Δs_ST_(Ω) values, respectively, are reported (in e^–^). These values are equivalent to minus the sum of the corresponding negative contributions. The atomic group decomposition is in terms of molecular subdomains defined as DCN = DCN1∪DCN2, THIO = THIO1∪THIO2, and M10A. All data in the Table are obtained using the DOCTRINE_SPIN code.

b *A* = ∑_[Δ*q*_CT_(Ω)]>0_Δ*q*_CT_(Ω) (or *B* = ∑_[Δ*s*_ST_(Ω)]>0_Δ*s*_ST_(Ω)). Equivalently, *A* (or *B*) is obtained as minus the sum of the only negative Δ*q*_CT_(Ω) (or Δ*s*_ST_(Ω)) values. See eq 23–24 for charge and 25–26 for spin.

**Figure 3:**
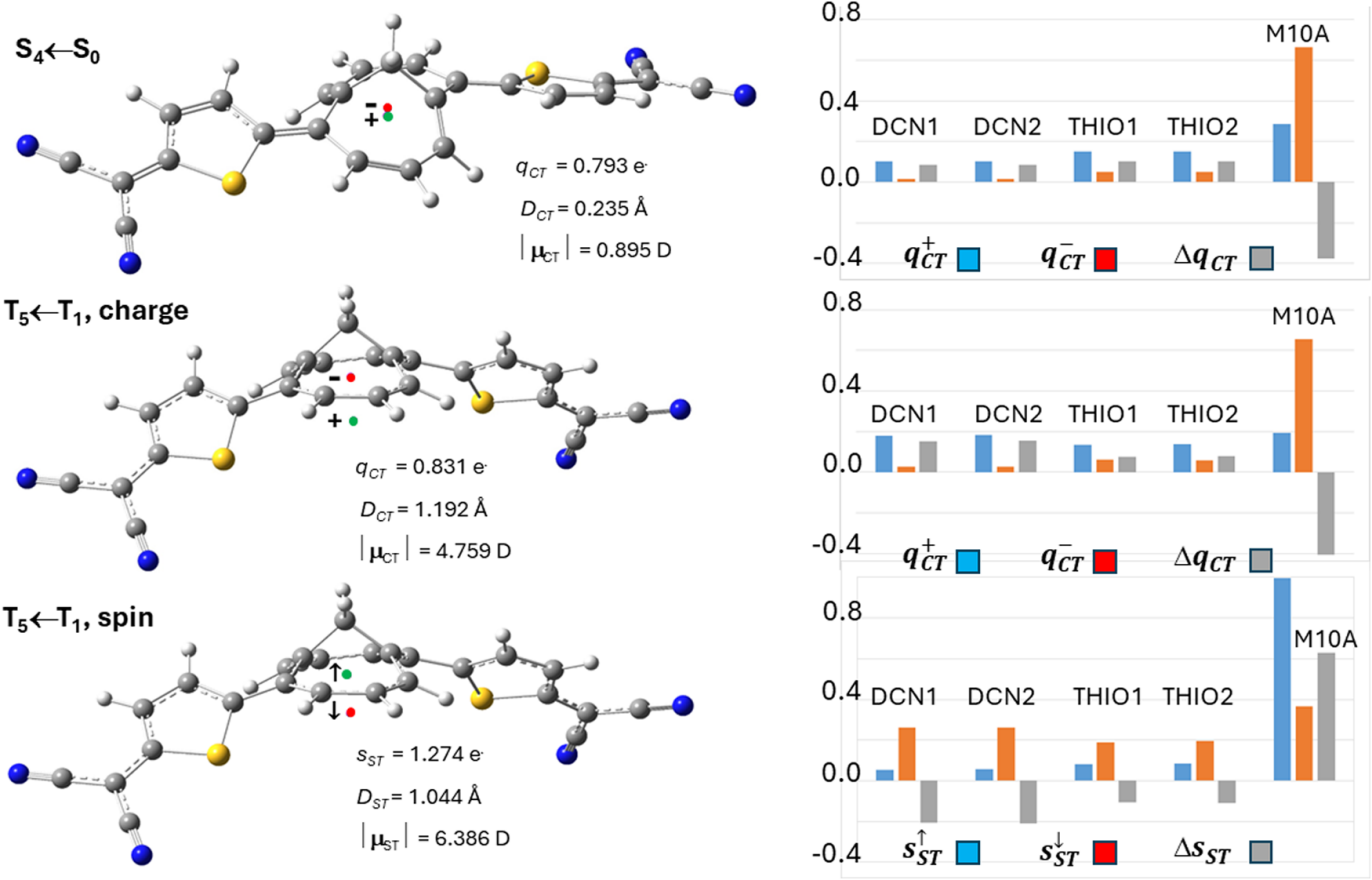
*TMTQ in nitromethane* (DFT and TD-DFT at the CAM-B3LYP/cc-pVDZ level): global charge transfer (CT) and spin transfer (ST) indexes (*left* panels) and atomic groups decomposition (*right* panels) of the transferred charge *q*_CT_ and spin *s*_ST_ upon excitation to the fourth singlet (S_4_) and triplet (T_5_) vertical excited states. The global CT indexes include the values of *q*_CT_ and *s*_ST_, the magnitude (in Debye, D) of the charge and spin dipole moment transfers between the ground and excited states, the CT and ST excitation lengths *D*_CT_ and *D*_ST_ (in Å), and the locations of the positive (spin-up) and negative (spin-down) centroids of the transferred charge or spin upon excitation (indicated in the left panels by green and red small spheres, respectively). The decompositions of *q*_CT_ and *s*_ST_ are shown both in terms of the *q*_CT_^+^(Ω) and of the *q*_CT_^–^(Ω) contributions (and *s*_ST_^↑^(Ω), *s*_ST_^↓^(Ω) for spin). For each subdomain Ω, the difference Δ*q*_CT_(Ω) (and Δ*s*_ST_(Ω) for spin) between the *q*_CT_^+^(Ω) and the *q*_CT_^–^(Ω) contributions (or between *s*_ST_^↑^(Ω) and *s*_ST_^↓^(Ω) for spin) is also shown. The TMTQ subdomains considered in the atomic group decomposition of the global CT and ST indexes in this and all subsequent figures are those defined in Figure 1.

**Figure 4:**
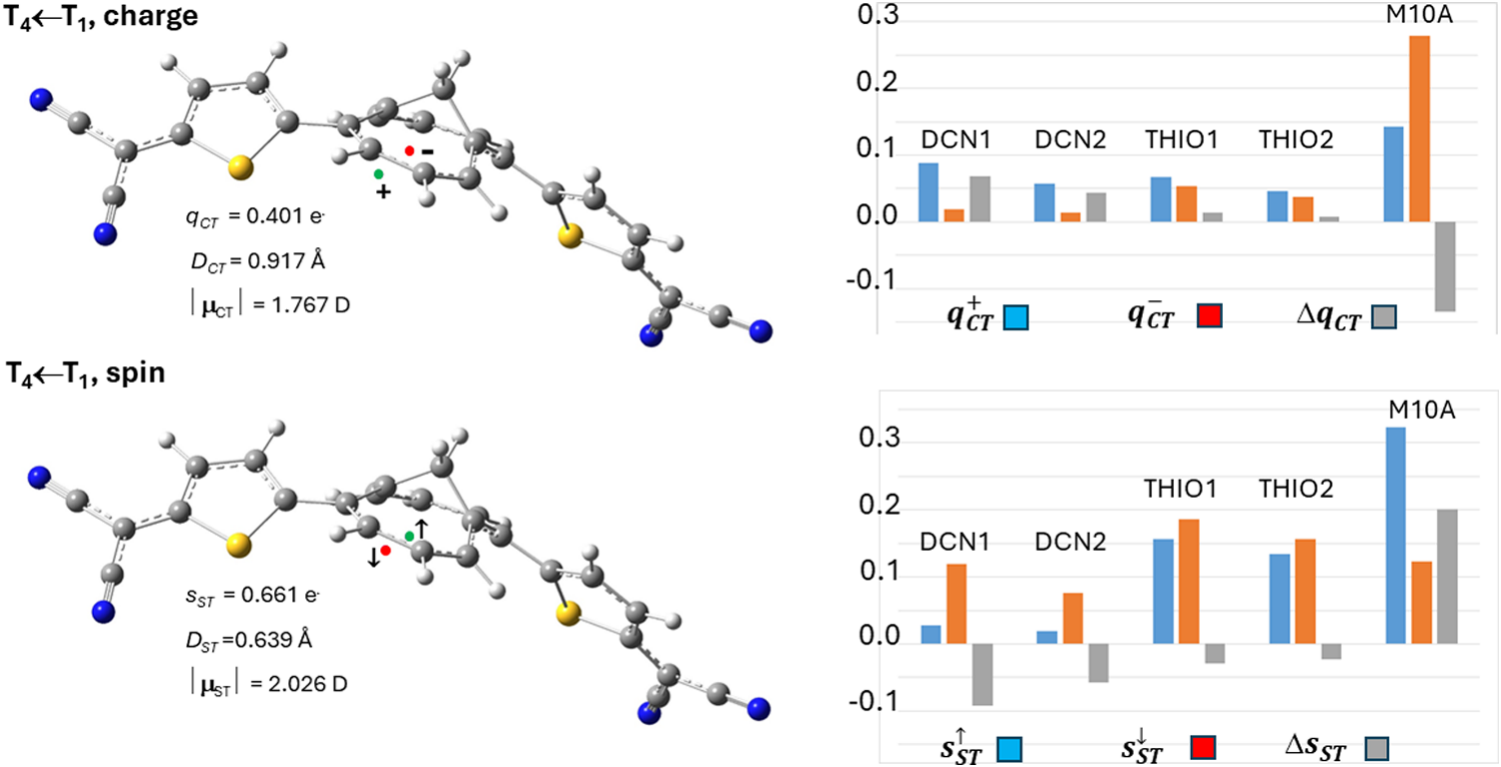
*TMTQ in nitromethane (DFT and TD-DFT at the CAM-B3LYP/cc-pVDZ level*): global charge transfer (CT) and spin transfer (ST) indexes (*left* panels) and atomic groups decomposition (*right* panels) of the transferred charge *q*_CT_ and spin *s*_ST_ upon excitation to the third triplet (T_4_) vertical excited state. For these excitations, asymmetries arise in the contributions from subdomains (THIO1 and THIO2, DCN1 and DCN2) that are chemically equivalent in the ground state. These asymmetries lead to significant shifts—compared to excited states without such asymmetry—in the positions of the positive (spin-up) and negative (spin-down) centroids of the transferred charge or spin upon excitation (shown in the left panels as green and red small spheres, respectively). Refer to the caption of Figure 3 for definitions of all quantities and symbols.

#### Global CT and ST Indexes

For both singlet and triplet states, the charge dipole moment change strongly depends on the specific excited state, with S_4_ and T_5_ exhibiting the largest values, 0.896 and 4.759 D, within the singlet and triplet excited state series, respectively. These values are even 1 order of magnitude larger, or significantly larger, relative to those excitations with the smallest dipole moment change in the corresponding series, namely S_1_ (0.050 D) and T_2_ (1.250 D). Especially for the singlet state excitations, the significant scatter in the values of the dipole moment change, ∥**μ**_CT_∥, results from extremely large relative variations in the excitation lengths *D*_CT_ (0.021-0.235 Å for singlet and 0.520–1.192 Å for triplet excited states), while the transferred electron charges, *q*_CT_, that multiplied by *D*_CT_ yield ∥**μ**_CT_∥, exhibit quite smaller relative variations (0.384–0.793 e^–^ for singlet and 0.401–0.831 e^–^ for triplet excited states). The smaller or larger *D*_CT_ value not only crucially determines the magnitude of the dipole moment change, especially for singlet states, but also explains why triplet states are characterized by significantly larger ∥**μ**_CT_∥ values. As reported above and in Table 2, the spin polarization allows for larger separations of the positive and negative poles of the transferred charge upon excitation, these separations being about 1 order of magnitude larger in the triplet states than in the corresponding singlet states. This means that the possibility to combine a charge transfer with a concomitant spin transfer strongly magnifies the dipole moment change thanks to a strongly enhanced separation of the positive and negative poles of the transferred charge, rather than to a significant increase of the transferred charge. In the triplet excitations, the spin transfer dipole moments are larger or of similar magnitude to the charge dipole moment changes, as a result of smaller excitation lengths and larger transferred spins, relative to the associated quantities for charge transfers. The spin transfer dipole moments range from 1.225 to 6.386 D and are ordered as are the charge dipole moment changes, with T_2_ exhibiting the lowest charge and spin dipole moment transfers, 1.250 and 1.225 D, respectively. The excitation with the largest charge dipole moment change, T_5_ (4.759 D), and excitation length (1.192 Å), corresponds to that with the largest spin dipole moment transfer (6.386 D) and spin excitation length *D*_ST_ (1.044 Å). Interestingly, the spin and charge dipole moments transfers are found to be essentially aligned but antiparallel, see Figure 3. Indeed, upon excitation, the centroid of the induced increase (decrease) of electron density, denoted by the positive (negative) pole, aligns with the centroid of the induced decrease (increase) of the spin density, denoted by the spin-down (spin-up) pole. Since T_5_ almost preserves the molecular symmetry (C_2_ axis), the poles of the positive (spin-up) and negative (spin-down) transferred charge and spin must lie on such an axis. The negative (spin-up) pole of the charge transfer (spin transfer) is located in the average plane of the annulenic ring, and the positive (spin-down) pole of the charge transfer (spin transfer) is far below this plane and lies opposite to the apical C4 carbon (for atom labeling, see Figure 2). Therefore, excitation of the ground triplet state to T_5_ involves a global electronic charge decrease and α-spin increase in the upper part of the molecule and a concomitant electronic charge increase and α-spin decrease (or, equivalently, β-spin increase), respectively, in its lower part (Figure 3). A chemical understanding of such an opposite displacement is easily provided by the charge and spin transfers decompositions in subdomain contributions (*see infra*). Comparison of the upper (S_4_ ← S_0_) and middle (T_5_ ← T_1_) left panels in Figure 3 pictorially shows the large *D*_CT_ enhancement made possible by the concomitant charge and spin transfers occurring in T_5_ ← T_1_. While the centroids of positive and negative transferred charges are very close to each other for S_4_ ← S_0_, *D*_CT_ = 0.235 Å, they are well separated from one another for T_5_ ← T_1_, *D*_CT_ = 1.192 Å. The bottom left panel of Figure 3 makes it evident that also the spin excitation length for T_5_ ← T_1_ is considerable and similar in magnitude, *D*_ST_ = 1.044 Å, to the associated charge excitation length, but with inverted poles as discussed earlier.

#### Subdomain Net Charge and Spin Changes Upon Excitation

We now analyze (Table 2) the changes in the net charge and spin upon excitation, Δ*q*_CT_(Ω) and Δ*s*_ST_(Ω) (eqs 17–19 and 20–22). The values of these quantities are also shown graphically in the right panels of Figure 3 for S_4_ ← S_0_ and T_5_ ← T_1_, and in Figure 4 for T_4_ ← T_1_. Also listed in Table 2 are the values of *A* and *B* (see eqs 23–24 and 25–26).

##### Singlet States: Subdomain Charge Transfer

For the singlet excitations, except for S_4_ ← S_0_, the values of *A* are small—about one order of magnitude lower than the transferred charge *q*_CT_ —because the positive and negative subdomain contributions, *q*_CT_^+^(Ω) and *q*_CT_^–^(Ω), are comparable. This indicates that all selected subdomains behave ambivalently during these excitations, with some regions contributing to the positive and others to the negative poles of the transferred charge. In contrast, the S_4_ ← S_0_ excitation behaves differently. For M10A, the contribution to the negative pole strongly prevails (*q*_CT_^–^(M10A) = 0.662 e^–^ *vs q*_CT_^+^(M10A) = 0.287 e^–^), while the opposite holds for DCN and THIO (*q*_CT_^–^ = 0.030 and 0.100 e^–^; *q*_CT_^+^ = 0.202 and 0.303 e^–^, respectively; see Figure 3, right panels). In this case, *A* is therefore large—approximately half the value of the transferred charge *q*_CT_—and all Δ*q*_CT_(Ω) are significant (−0.375, 0.171, and 0.205 e^–^ for M10A, DCN, and THIO, respectively).

The largest dipole-moment change and excitation length for the singlet excitations occur when (*a*) the subdomains maximize their electron-withdrawing or electron-donating behavior, thereby minimizing their ambivalent character, and (*b*) the excitation leads to a state with the largest contribution from the Baird-aromatic (8π-electron)/polar structure **c**. Indeed, S_4_ ← S_0_ is characterized by the strongest M10A negative charge transfer, Δ*q*_CT_(M10A), the largest decrease in M10A electron population (*i.e.*, the most positive Δ*q*_M10A_ = 0.349 e^–^), and the largest decrease in both the FLU index (Δ_FLU_ = −0.019) and the DI standard deviation (Δstd_DI_= −0.110; see Table 1).[Fn fna]

##### Triplet States: Subdomain Charge Transfer

Excitations from the ground triplet state show both analogies and notable differences relative to the corresponding singlet excitations. The largest value of *A* (0.463 e^–^) is slightly more than half of the transferred charge *q*_CT_ (0.831 e^–^) and, as in the S_*n*_ ← S_0_ series, occurs for the fourth excited state (T_5_). However, other excited states also display significant *A* values (0.168–0.240 e^–^), corresponding to *q*_CT_ values between 0.401 and 0.553 e^–^. This indicates that the positive (*q*_CT_^+^(Ω)) and negative (*q*_CT_^–^(Ω)) subdomain contributions to *q*_CT_ differ substantially for all excitations in the T_*n*_ ← T_1_ series, unlike the S_*n*_ ← S_0_ series, where this occurs only for S_4_ ← S_0_.

The possibility of simultaneous charge and spin transfer allows the selected subdomains in the triplet excitations to behave much less ambivalently than in the singlet series. In all excitations except T_6_ ← T_1_, the contribution to the negative pole is strongly dominated by M10A, with Δ*q*_CT_(M10A) showing a negative peak (−0.463 e^–^) at T_5_ ← T_1_. These values correspond to −*A*, since for both THIO and DCN, the positive contributions *q*_CT_^+^ prevail, resulting in consistently positive Δ*q*_CT_ values.

As in the singlet series, the largest charge dipole moment change and excitation length occur for the fourth excitation (T_5_), when (a) the subdomains maximize their electron-withdrawing or -donating roles (minimizing ambivalence), and (b) the strongest M10A negative charge transfer, Δ*q*_CT_(M10A), and the largest decrease in M10A electron population (*i.e.*, the most positive Δ*q*_M10A_ = 0.432 e^–^, Table 1) occur. However, unlike S_4_ ← S_0_, the T_5_ ← T_1_ excitation does not exhibit the largest decrease in the FLU index within the series—the largest (though still small) decrease occurs at T_3_ ← T_1_ (ΔFLU = −0.005)—nor does it show a reduction in the delocalization index standard deviation, which remains essentially unchanged (Δstd_DI_= −0.000) relative to the ground triplet state.

This behavior is consistent with the triplet TMTQ system being best described by the diradical/Hückel-aromatic (10π-electron) structure **b**, with a minor contribution from the Baird aromatic (8π-electron)/polar structure **c**. An increased contribution from structure **c** upon excitation—evidenced by the large negative Δ*q*_CT_(M10A) and large positive Δ*q*_M10A_—does not imply enhanced aromaticity of the annulenic moiety, since both structures **b** and **c** are aromatic.

In the single case of the T_6_ ← T_1_ excitation, Δ*q*_CT_(M10A) is small and positive (0.010 e^–^), while Δ*q*_CT_(DCN) is largely positive (0.227 e^–^) and Δ*q*_CT_(THIO) largely negative (−0.240 e^–^), indicating a qualitatively different excitation. This transition is characterized by a slight decrease, rather than a significant increase, in the weight of the Baird-aromatic (8π- electron)/polar structure **c** upon excitation (Δ*q*_M10A_ = −0.008, in contrast with the large positive values found for the other triplet excitations; see Table 1). Since both TMTQ structures **b** and **c** are aromatic, T_6_ ← T_1_ displays only small decreases in both the delocalization index standard deviation and the FLU index relative to the ground triplet state (Δ*std*_DI_ = −0.012 and Δ_FLU_ = −0.007).

##### Triplet States: Subdomain Spin Transfer

We now examine the spin-related features of the triplet series. The transferred spin, *s*_ST_, is consistently much larger (0.661/1.274 e^–^) than the transferred charge, *q*_CT_ (0.401/0.831 e^–^), for all excitations—often by as much as a factor of 2. The largest *B* value (0.629 e^–^) is nearly half of the transferred charge *q*_CT_ (1.274 e^–^) and, as in the case of the charge-related quantity *A*, occurs for the fourth excitation (T_5_). Significant *B* values (0.188/0.500), corresponding to *s*_ST_ values between 0.661 and 1.071 e^–^, also characterize the other excited states. As with *q*_CT_, the positive and negative subdomain contributions to *s*_ST_ differ substantially for all excitations in the series. Thus, for spin as for charge, the possibility of simultaneous charge and spin transfer allows the selected subdomains in the triplet excitations to behave far less ambivalently than in the singlet series. For all excitations except T_6_ ← T_1_, the spin-up contribution is strongly dominated by M10A: Δ*s*_ST_(M10A) peaks at 0.629 e^–^ for T_5_ ← T_1_.

As for charge, the largest spin transfer dipole moment and the longest spin excitation length occur for the fourth excitation (T_5_), where (a) the subdomains maximize their propensity either to increase or decrease spin (minimizing spin ambivalence), and (b) both the strongest M10A spin up transfer, Δ*s*_ST_(M10A), and the largest increase in M10A spin population (i.e., the most positive Δs_M10A_ = 0.628 e^–^, Table 1) are observed. This behavior further supports the enhanced contribution of the Baird- aromatic (8π-electron)/polar structure **c** in the T_5_ ← T_1_ excitation. The increased weight of this structure—characterized by a ^3^M10A^2+^ moiety—is reflected in a large positive spin-transfer contribution from M10A, Δ*s*_ST_(M10A), a large increase in its spin population Δ*s*_M10A_, and, as mentioned previously, a large negative charge transfer contribution Δ*q*_CT_(M10A) together with an increase in its positive charge, Δ*q*_M10A_. By contrast, the THIO groups, and even more markedly the DCN groups, display the opposite behavior.

In the unique case of the T_6_ ← T_1_ excitation, Δ*s*_ST_(M10A) becomes small and negative (−0.049 e^–^), whereas Δ*s*_ST_(THIO) is strongly positive (0.501 e^–^) and Δs_ST_(DCN) strongly negative (−0.452 e^–^), underscoring the markedly different nature of this excitation also in terms of spin.

This transition is therefore characterized by a slight decrease—rather than a significant increase—in the weight of the Baird-aromatic (8π-electron)/polar structure **c** upon excitation (Δ*s*_M10A_ = −0.049, contrasting sharply with the large positive values for the other triplet excitations; see Table 1). The most important feature revealed by the spin data is that the DCN groups lose about half of their spin, which becomes localized instead on the THIO groups (from 0.769 e^–^ in T_1_ to 1.270 e^–^ in T_6_), particularly on the sulfur atoms.

##### Symmetry Breaking

The T_6_ ← T_1_ transition is also marked by significant symmetry breaking between the left and right TMTQ molecular moieties (Figure 1), evidenced by the differing behavior of the two DCN and the two THIO groups: one side of the molecule undergoes substantially larger charge and spin transfers than the other. For example, Δ*q*_CT_(Ω) and Δ*s*_ST_(Ω) differ by 0.055 e^–^ and 0.084 e^–^ between the two DCN groups and by 0.060 e^–^ and 0.109 e^–^ between the two thiophene groups, respectively.

We end this section by briefly discussing the distinctive features of the T_4_ ← T_1_ excitation shown in Figure 4. Although qualitatively similar in nature to the T_5_ ← T_1_ transition (as are T_2_ ← T_1_ and T_3_ ← T_1_), it is unique among the three for the pronounced asymmetry in the contributions of the two DCN and THIO groups. This is readily appreciated from the asymmetric Δ*q*_CT_(Ω) and Δs_ST_(Ω) values, and even more so from their component *q*_CT_^+^(Ω) and *q*_CT_^–^(Ω) contributions, between DCN1 and DCN2 or THIO1 and THIO2 (left panels of Figure 4). This asymmetry—particularly significant for the spin properties of the DCN groups—is nearly absent in the T_5_ ← T_1_ transition (compare the left panels of Figure 3 and Figure 4). The T_4_ ← T_1_ asymmetry also leads to a markedly asymmetric positioning of the charge- and spin-transfer centroids. As shown in Figure 4, these centroids no longer lie along the molecular C_2_ axis as in Figure 3; instead, they lie nearly within the plane of the M10A ring and are significantly displaced from its center.

### Charge- and Spin-Transfer Dipole Moments and Their Atomic Groups Decomposition (According to Eqs 39–40 and 41–42)

Figures 5 and 6 and Table S5 display the atomic groups decomposition of the norm of the charge- and spin-transfer dipole moments, ∥**μ**_CT_∥ and ∥**μ**_ST_∥, for the singlet (Figure 5) and triplet (Figure 6) excited states of TMTQ in nitromethane. The μ_CT_^+^(Ω) and the μ_CT_^–^ (Ω) contributions (and μ_ST_^↑^(Ω) and μ_ST_^↓^(Ω) for spin) to ∥**μ**_CT_∥ and ∥**μ**_ST_∥ are calculated according to eqs 39–40 (charge) and 41–42 (spin), which retain only the dependence of ∥**μ**_CT_∥ on *q*_CT_ and of ∥**μ**_ST_∥ on *s*_ST_. In Figures 5 and 6, the molecular subdomains Ω correspond to those defined in Figure 1, while in Table S5, contributions from DCN1 and DCN2 and from THIO1 and THIO2 are grouped.

**Figure 5:**
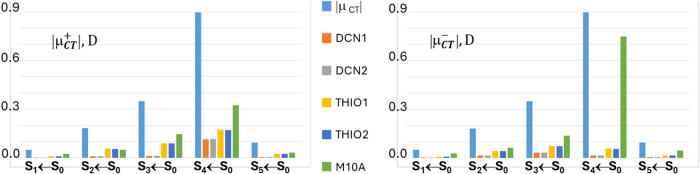
Atomic group decomposition of the norm of the charge-dipole moment change, ∥**μ**_CT_∥, (Debye, D), for the first five singlet vertical excited states of TMTQ in nitromethane, computed at the DFT and TD-DFT CAM-B3LYP/cc-pVDZ level. For each excitation, the total dipole moment change is shown as an azure bar. The individual μ_CT_^+^(Ω) and μ_CT_^–^(Ω) contributions to ∥**μ**_CT_∥ are shown for the molecular subdomains defined in Figure 1. Contributions are computed according to eqs 39–40. Very small contributions are barely visible in the plot (numerical values are provided in Table S5).

**Figure 6:**
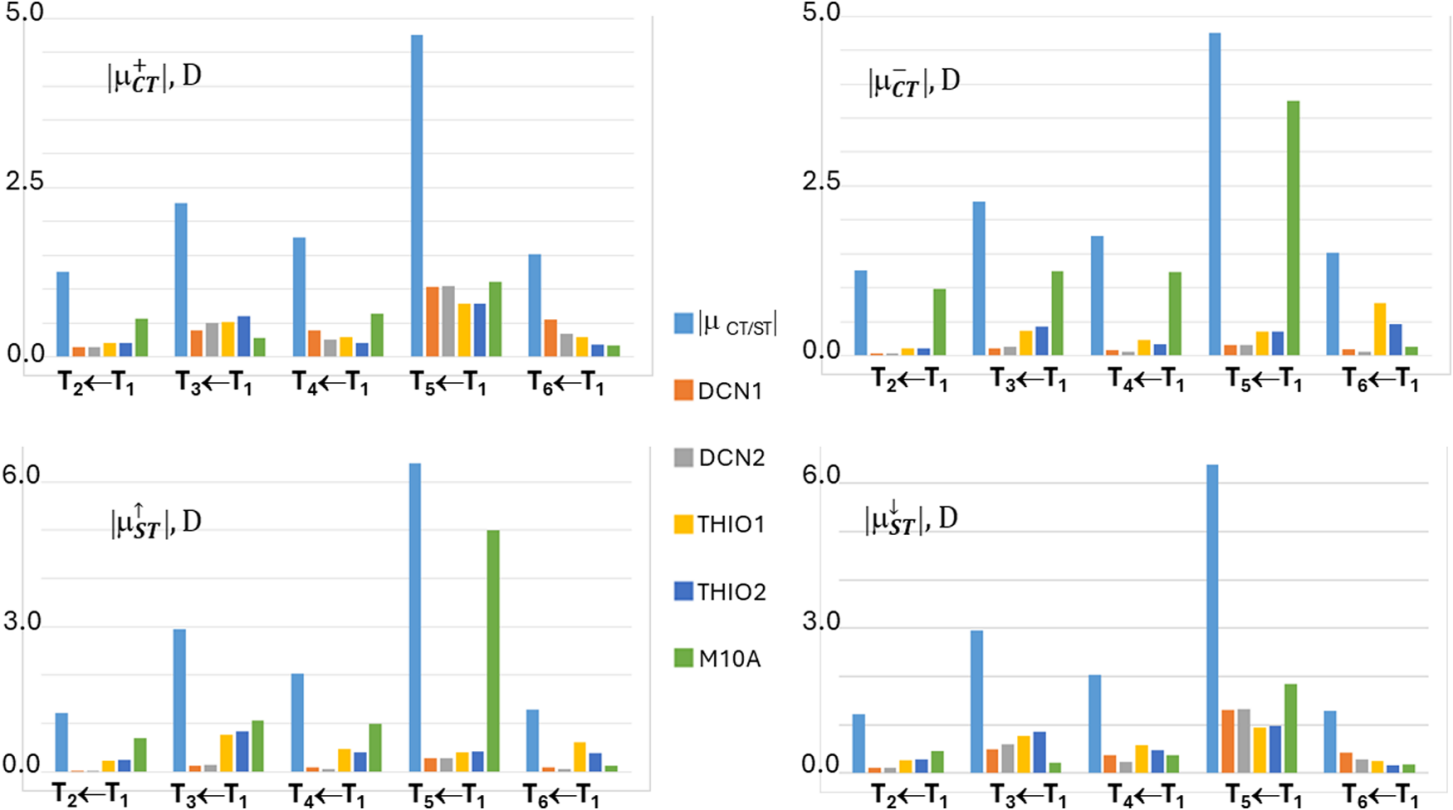
Atomic group decomposition of the norms of the charge- and spin-transfer dipole moments, ∥**μ**_CT_∥ and ∥**μ**_ST_∥ (Debye, D), for the first five triplet vertical excited states of TMTQ in nitromethane (DFT and TD-DFT CAM-B3LYP/cc-pVDZ level). For each excitation, total charge- and spin-transfer dipole moments are displayed as azure bars. The individual μ_CT_^+^(Ω) and μ_CT_^–^(Ω) contributions (and μ_ST_^↑^(Ω) and μ_ST_^↓^(Ω) for spin) are shown for the molecular subdomains defined in Figure 1. Contributions are computed according to eqs 39–40 (charge) and eqs 41–42 (spin). Very small contributions are difficult to distinguish visually; numerical values are reported in Table S5.

#### Singlet States: Charge- and Spin-Transfer Dipole Moments Decomposition

Table S5 and Figure 5 indicate that, for the singlet series, the THIO groups provide the largest μ_CT_^+^(Ω) contribution to the charge-dipole moment change for all excitations except S_1_ ← S_0_ whose ∥**μ**_CT_∥ is nearly negligible. The M10A group contributes comparably, particularly in S_4_ ← S_0_, the singlet excitation displaying the largest dipole moment change and the only one for which DCN groups also contribute substantially. The decomposition of ∥**μ**_CT_∥ in terms of μ_CT_^–^(Ω) is, as expected, markedly different. The S_4_ ← S_0_ dipole moment change is dominated by μ_CT_^–^(M10A), accounting for 83.5% of the total. The remaining singlet excitations show comparable μ_CT_^–^(Ω) contributions from THIO and M10A, whereas DCN contributions remain modest for all states. The decomposition of ∥**μ**_CT_∥ into μ_CT_^+^(Ω) and μ_CT_^–^(Ω) clearly reflects the decomposition of *q*_CT_ into *q*_CT_^+^(Ω) and *q*_CT_^–^(Ω) (eqs 39–40) and analogous considerations apply to spin quantities (eqs 41–42). As Figure 5 illustrates, singlet excitations do not lead to any appreciable molecular symmetry breaking.

#### Triplet States: Charge- and Spin-Transfer Dipole Moments Decomposition

##### Charge Dipole Moment Analysis

Table S5 and Figure 6 highlight pronounced differences between the singlet and triplet manifolds, not only in the magnitude of the dipole moment changes, but also in their atomic group decomposition. For μ_CT_^+^(Ω), significant contributions arise from all groups for all triplet excitations. The DCN groups, which become especially relevant when simultaneous charge/spin rearrangements are possible, play a major role in reconstructing ∥**μ**_CT_∥ from μ_CT_^+^(Ω) for the three highest excitations. In particular, μ_CT_^+^(DCN) contributes 43.8% of the largest charge-dipole moment in the series (4.759 D, T_5_ ← T_1_). For T_2_ ← T_1_, the largest μ_CT_^+^(Ω) contribution comes from M10A (45,1%) and for T_3_ ← T_1_ from THIO (48,8%).

The decomposition in terms of μ_CT_^–^(Ω) is strikingly different. The largest dipole moment change (T_5_ ← T_1_) is almost entirely (78.9%,) determined by μ_CT_^–^(M10A), reflecting the dominant *q*_CT_^–^(M10A) contribution (Table 2), i.e., the strong decrease of the M10A electron population, Δ*q*_M10A_. In all excitations except T_6_ ← T_1_, μ_CT_^–^(M10A) outweighs contributions from the other groups; in T_6_ ← T_1_, the largest contribution arises from THIO.

In contrast to the singlet series, a clear molecular symmetry breaking is observed for the triplets, both between DCN1/DCN2 and THIO1/THIO2, especially for T_3‑4_ and T_6_ (Figure 6).

##### Spin-transfer Dipole Moment Analysis

Spin-transfer dipole moments (Table S5 and Figure 6, bottom panels) display a distinct pattern in terms of their μ_ST_^↑^(Ω) contributions. The largest spin-transfer dipole moment (6.386 D) occurs in T_5_ ← T_1_ and is dominated by μ_ST_^↑^(M10A) (78,1%). The second largest (2.947 D, T_3_ ← T_1_) receives substantial contribution from both THIO (54,6%) and M10A (36%). For T_4_ ← T_1_, these two groups contribute similarly, and a similar trend is observed for T_2_ ← T_1_. As expected from the dominant *s*_ST_^↑^ (THIO) contribution to *s*_ST_ (Table 2), ∥**μ**_ST_∥ for (T_6_ ← T_1_) is largely determined by μ_ST_^↑^(THIO).

The μ_ST_^↓^(Ω) decomposition shows a different pattern. For the three first excited states, μ_ST_^↓^(THIO) provides the largest contribution. In the remaining states, μ_ST_^↓^(DCN) dominates. Although T_5_ ← T_1_ exhibits the largest overall spin-transfer dipole moment, its μ_ST_^↓^(Ω) components are similar across the three groups (41,1%, 30,1%, 28,8% for DCN, THIO, M10A), indicating a strongly concerted excitation involving a decrease of α-spin (equivalently an increase of β-spin).

As with charge, signatures of molecular symmetry breaking are observed for spin, depending on the excitation number and on whether μ_ST_^↑^(Ω) or μ_ST_^↓^(Ω) is examined (Figure 6, bottom panels).

### Decomposition of the CT Excitation Length into Atomic Group Subdomains Contributions (Eqs 29–30)

Figure 7 and Table S6 show the atomic group decomposition of the charge-transfer (CT) and spin-transfer (ST) excitation lengths, ***D***_CT_ and ***D***_ST_, for the first five singlet and triplet states. Only the *z* component, **d**_CT,*z*_^Ω^ or **d**_ST,*z*_^Ω^, of each atomic group’s contribution to the excitation length vector ***D***_CT_ or ***D***_ST_ is reported, as the *z* axis (the C_2_ molecular axis pointing toward the apical M10A carbon) is nearly collinear with ***D***_CT_ and ***D***_ST_ in most cases.

**Figure 7:**
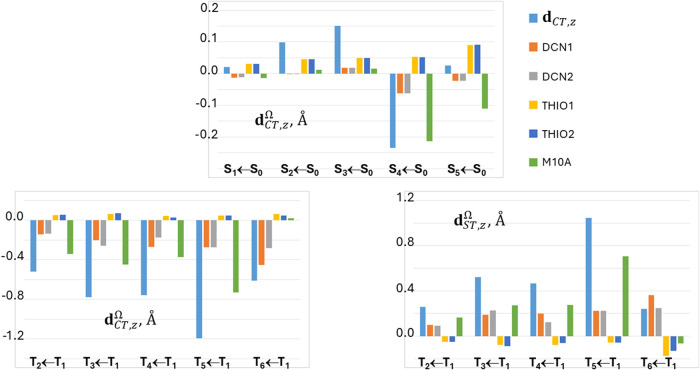
Atomic group decomposition of the charge-transfer (CT) and spin-transfer (ST) excitation lengths, ***D***_CT_ and ***D***_ST_, for the singlet and triplet vertical excited states of TMTQ in nitromethane, computed using DFT and TD-DFT at the CAM-B3LYP/cc-pVDZ level. Only the *z* component, **d**_CT,*z*_^Ω^ or **d**_ST,*z*_^Ω^ , is shown, as the *z* axis is nearly collinear with the total excitation length vector, ***D***_CT_ (or ***D***_ST_), for most excitations. Numerical values and additional details are provided in Table S6.

The degree of collinearity is quantified by α_CT,*z*_ or α_ST,*z*_ , i.e., the *z*-component of the direction cosine of the **R** ^+^ – **R** ^–^ (or **S** ^↑^ – **S** ^↓^) vector (eqs 33–34). Deviations of α from ±1 indicate departures from perfect collinearity or anti-collinearity. Values of ***g***_CT,*z*_^Ω^ (or ***g***_ST,*z*_^Ω^), defined as the ratio of **d**_CT,*z*_^Ω^ (or **d**_ST,*z*_^Ω^) to the total excitation length *D*_CT_ (or *D*_ST_), are also reported (eqs 31–32). These dimensionless quantities indicate the relative extent to which a given atomic group contributes to or opposes the direction and magnitude of the excitation length. For each excitation, the largest absolute values of **d**_CT,*z*_^Ω^ and ***g***_CT,*z*_^Ω^ (or **d**_ST,*z*_^Ω^ and ***g***_ST,*z*_^Ω^ ) are shown in bold. In Figure 7, the total CT and ST excitation lengths are shown as azure bars. As in Table S5, DCN1/2 and THIO1/2 contributions are summed to yield DCN and THIO.

#### Analysis of Singlet Excitation Lengths

All singlet excitation lengths are oriented along the *z* axis (positive sign), except S_4_ ← S_0_, which uniquely points in the opposite direction (α_CT,*z*_ = −0.996) and also exhibits the largest dipole moment change within the singlet series. All excitation vectors are nearly collinear with the *z* axis, except S_5_ ← S_0_, which deviates significantly (α_CT,*z*_ = 0.604).

The unusual orientation of S_4_ ← S_0_ arises because THIO is not the dominant contributor (***g***_CT,*z*_^THIO^ = 0.439) and both DCN and M10A strongly oppose the total excitation length (***g***_CT,*z*_^DCN^ = −0.530, ***g***_CT,*z*_^M10A^ = −0.905). The **d**_CT,*z*_^Ω^ value for S_4_ ← S_0_ is the largest among all singlet **d**_CT,*z*_^Ω^ contributions (Table S6, Figure 7).

Inspection of **d**_CT,*z*_^Ω^ or ***g***_CT,*z*_^Ω^ reveals that the contributions often exceed the magnitude of ***D***_CT_ itself—sometimes by factors of 3 to 4 (*e.g.*, THIO in S_5_ ← S_0_). This reflects the strongly compensating nature of the contributions, which is immediately evident from the group-resolved analysis in Figure 7.

#### Triplet Excitation Lengths

Triplet excitations exhibit a markedly different behavior due to the possibility of simultaneous charge and spin rearrangements. ***D***_CT_ values are typically 1 order of magnitude larger than those of the singlets and differ in alignment, sign, and decomposition.

All triplet excitations are oppositely directed to the *z* axis, with two of them (T_2_ and T_5_) perfectly antiparallel to it and the other three showing an appreciable misalignment, in particular T_4_ ← T_1_ (α_CT,*z*_ = −0.824). The dominant THIO group contribution seen in singlets is consistently small in the triplet manifold. For T_2_ and T_5_, **d**_CT,*z*_^M10A^ is the largest contribution, but comparable to that from DCN, while for T_3_ and T_4_, the DCN contribution dominates. In these four states, M10A and DCN reinforce (rather than oppose) each other, revealing their cooperative nature. In contrast, T_6_ ← T_1_ is dominated by DCN, while THIO and M10A oppose the total length.

Spin-transfer excitation lengths *D*_ST_ are always smaller but comparable to *D*_CT_, especially for T_5_ ← T_1_, which has the largest values in the series. Unlike ***D***_CT_, the ***D***_ST_ vector is aligned along the positive *z* axis, perfectly parallel for T_5_ and partially misaligned for the other excitations, especially T_4_ ← T_1_ (α_ST,*z*_ = 0.730).

As with ***D***_CT_, the *z*-component of ***D***_ST_ is dominated by DCN and M10A contributions, with DCN generally somewhat larger except for T_5_ ← T_1_, where M10A surpasses DCN (0.705 vs. 0.449 Å). THIO consistently opposes ***D***_ST_ and does so strongly for T_6_ ← T_1_, where DCN makes the dominant positive contribution (***g***_ST,*z*_^DCN^ = 2.444 *vs**g***_ST,*z*_^M10A^ = −1.216).

In sharp contrast with the singlet series, molecular symmetry breaking is prominent in both charge- and spin-transfer excitation lengths for all triplet excitations except T_5_ ← T_1_, which uniquely combines the largest *D*_CT_ and *D*_ST_ values with perfect electronic excitation symmetry.

### Decomposition of the μ_CT_ and μ_ST_ Vectors into Atomic Group Subdomain Contributions (According to Eqs 37–38)

Table S7 and Figures 8, 9 show and detail the decomposition of the *z*-component of the charge- and spin-transfer dipole moment vectors, **μ**_CT_ and **μ**_ST_, into *intra*-subdomain (μ_CT_^intra^ or μ_ST_^intra^) and *inter*-subdomain (μ_CT_^inter^ and μ_ST_^inter^) contributions. The decomposition of the **μ**_CT_ and **μ**_ST_ vectors is obtained through eqs 37–38, which—unlike what is reported in Figures 5 and 6 and Table S5—account for the dependence of **μ**_CT_ (and **μ**_ST_) on both *q*_CT_ and *D*_CT_ (*q*_ST_ and *D*_ST_).

**Figure 8:**
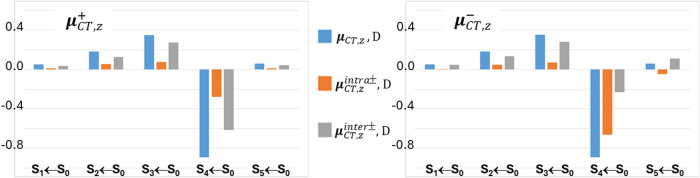
Decomposition of the *z*-component of the charge-transfer (CT) dipole moment vector, **μ**_CT_, (in Debye, D), into *intra*-subdomain (μ_CT_^intra^) and *inter*-subdomain (μ_CT_^inter^) contributions, as defined in eq 37. Results refer to the first five singlet vertical excited states of TMTQ in nitromethane, computed using DFT and TD-DFT at the CAM-B3LYP/cc-pVDZ level. Only the *z*-component is shown, since the *x* and *y* components are negligible for most excited states. Intra- and inter-subdomain contributions to **μ**_CT_ are shown as derived either from the *M*_CT_^*z*,+^ or *M*_CT_^*z*,–^ matrices, whose elements sum to **μ**_CT,*z*_^+^ and **μ**_CT,*z*_^–^ (eq 35–36).

**Figure 9:**
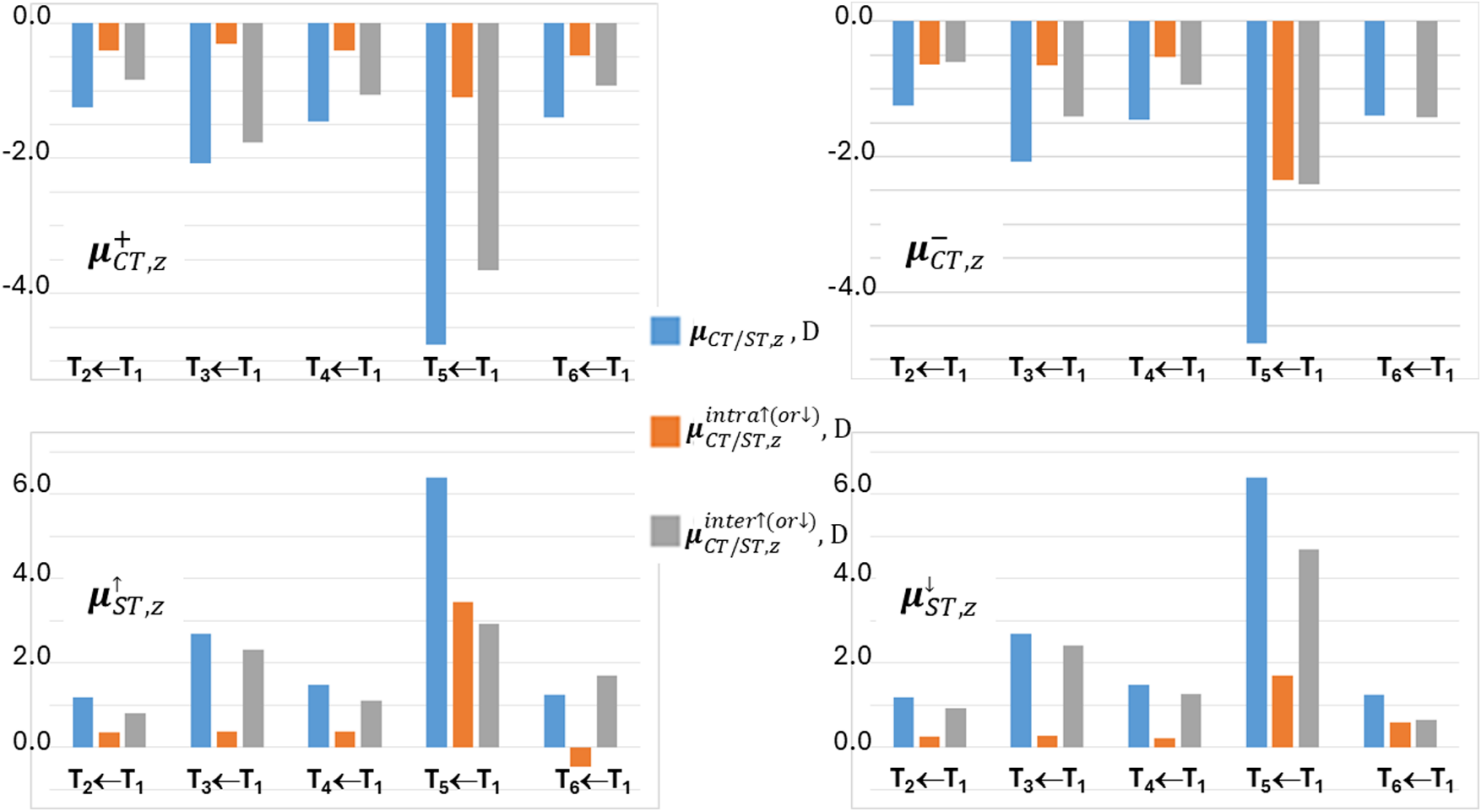
Decomposition of the *z*-component of the charge-transfer (CT) and spin-transfer (ST) dipole moment vectors, **μ**_CT_ and **μ**_ST_ (in Debye, D), into *intra*-subdomain (μ_CT_^intra^ or μ_ST_^intra^) and *inter*-subdomain (μ_CT_^inter^ or μ_ST_^inter^) contributions, according to eq 37 (for CT) and eq 38 (for ST). Results refer to the first five triplet vertical excited states of TMTQ in nitromethane, computed using DFT and TD-DFT at the CAM-B3LYP/cc-pVDZ level. Only the *z*-component is reported, since the *x* and *y* components are negligible for most excited states. Intra- and inter-subdomain contributions are derived from the *M*_CT_^*z*,+^ or *M*_CT_^*z*,–^ matrices (and from the *M*_ST_^*z*,↑^ or the *M*_ST_^*z*,↓^ matrices), whose elements sum to **μ**_CT,*z*_^+^ and **μ**_CT,*z*_^–^ (or **μ**_ST,*z*_^↑^ and **μ**_ST,*z*_^↓^) as given in eqs 35–36

Results are shown for the first five singlet (Table S7 and Figure 8) and triplet (Table S7 and Figure 9) vertical excited states of TMTQ in nitromethane, and only the *z*-component is reported, since the *x* and *y* components are negligible for most excited states. Intra- and inter-subdomain contributions to **μ**_CT_ (**μ**_ST_) are listed as derived either from the *M*_CT_^z,+^ or *M*_CT_^z,–^ matrices (from the *M*_ST_^z,↑^ or *M*_ST_^z,↓^ matrices), whose elements sum to the **μ**_CT,z_^+^ and **μ**_CT,z_^–^ (or **μ**_ST,z_^↑^ and **μ**_ST,z_^↓^) components of the charge- (or spin-transfer) dipole moment vectors (eqs 35–36). Note also that, in general, the values of the intra- and inter-subdomain contributions to **μ**_CT_ (**μ**_ST_) depend on whether they are calculated using *q*_CT_^+^ or *q*_CT_^–^(*s*_ST_^↑^ or *s*_ST_^↓^), whereas their sum—that is, **μ**_CT_ (or **μ**_ST_)—does not.

In the case of singlet excitations, intra- and inter-subdomain contributions always concur to the observed **μ**_CT,*z*_ value, except for S_5_ ← S_0_, for which the reconstruction using *q*_CT_^–^ is overestimated by almost a factor of 2, by the inter-subdomain contribution. For all excitations except S_4_ ← S_0_, the inter-subdomain contribution largely prevails over the intra-subdomain one in determining **μ**_CT,*z*_, regardless of whether the decomposition is performed using *q*_CT_^+^ or *q*_CT_^–^.

For S_4_ ← S_0_—*i.e.*, the excitation with the largest |**μ**_CT,*z*_| value and the only one in which the charge-transfer dipole moment is antiparallel to the *z-*axis—the intra-subdomain term accounts for 74% of **μ**_CT,z_ when the decomposition is performed using *q*_CT_^–^, whereas using *q*_CT_^+^ it is the inter-subdomain contribution (68.8% of **μ**_CT,*z*_) that predominates, as in all other cases.

Thus, the excitation with the largest |**μ**_CT,*z*_| is characterized by a dominant inter-subdomain contribution when the reconstruction of |**μ**_CT,*z*_| is based on ρ^+^, whereas the intra-subdomain contribution dominates when the reconstruction is based on ρ^–^.

#### Triplet Excitations

For the triplet excitations, similar considerations apply to the decomposition of **μ**_CT,*z*_. As noted earlier, **μ**_CT,*z*_ is always directed opposite to the *z*-axis for the triplet states and, as for the singlets, the inter-subdomain contribution generally dominates when *q*_CT_^+^ is used to reconstruct **μ**_CT,*z*_. Conversely, using *q*_CT_^–^, the intra-subdomain contribution becomes generally larger—except for T_6_ ← T_1_—and even comparable in magnitude to the inter-subdomain contribution for T_2_ ← T_1_ and T_5_ ← T_1_. Also note that for all excitations except T_6_ ← T_1_, the intra-subdomain contribution to **μ**_CT_ is larger in magnitude (often more than twice as large) when calculated using *q*_CT_^–^ instead of *q*_CT_^+^.

The spin-transfer dipole moment **μ**_ST_ is oriented along the +*z*-axis, with contributions overwhelmingly dominated by the inter-subdomain term, both when using *s*_ST_^↑^ and *s*_ST_^↓^. A notable exception is the T_5_ ← T_1_ excitation, which has the largest **μ**_ST,*z*_ value and shows a slightly larger intra-subdomain contribution when using *s*_ST_^↑^, whereas the inter-subdomain contribution dominates when using *s*_ST_^↓^.

#### General Considerations

Overall, for both singlet and triplet excitations, it appears that the largest contributions to the charge and spin-transfer dipole moments arise from mixed (inter-subdomain) terms, where the charge- (or spin-) transfer in one subdomain couples with the excitation-charge (or -spin) transfer length of a different subdomain. Accordingly, intra-subdomain contributions to the charge- or spin-transfer dipole moments are generally less effective than those delocalized across two subdomains. However, important exceptions occur—particularly for the excitations with the largest charge- or spin-transfer dipole moment within each series, and especially when considering the decomposition based on charge-decrease or spin-decrease contributions.

### Subdomain Charge- and Spin-Transfer Dipole Moment Matrices

In the previous section, we discussed the decomposition of **μ**_CT_ and **μ**_ST_ into their intra- and inter-subdomain contributions. Such a decomposition is a *condensed* representation of the complete information contained in the *M*_CT_^*i*,+^ and *M*_CT_^*i*,–^ matrices (or *M*_ST_^*i*,↑^ and *M*_ST_^*i*,↓^) given in eqs 37–38. As examples, Tables 3 and S8 list the matrix elements of *M*_CT_^*z*,+^ and *M*_CT_^*z*,–^, and of *M*_ST_^*z*,↑^ and *M*_ST_^*z*,↓^, for the S_4_ ← S_0_ and T_5_ ← T_1_ vertical excitations of TMTQ. Matrix elements corresponding to the *x* and *y* components of **μ**_CT_ and **μ**_ST_ are not reported, since they are orders of magnitude smaller than those of the *z* component, because the charge- and spin-excitation length vectors ***D***_CT_ and ***D***_ST_ are almost antiparallel and parallel to the *z-*axis, respectively, for these two excitations (see Table S6).

**Table 3:**
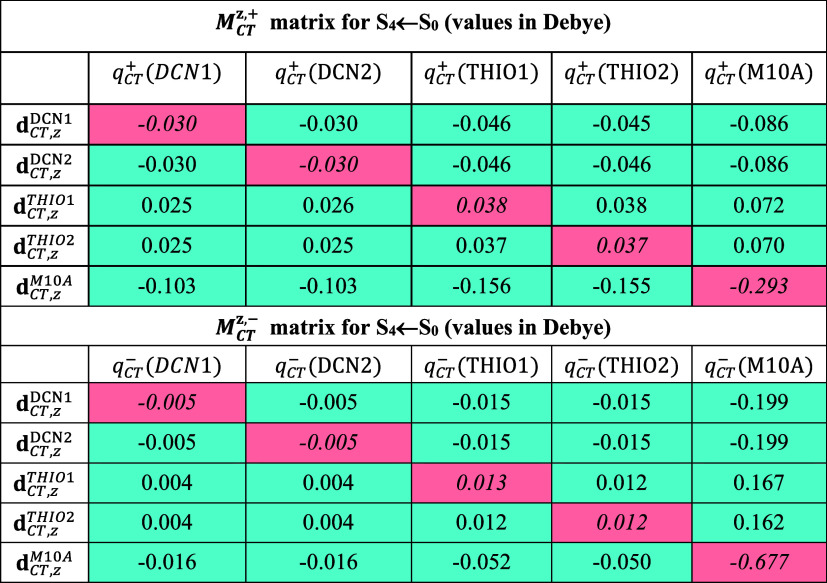
Matrix Elements (in Debye, D) of *M*_CT_^*z*,+^ and *M*_CT_^*z*,–^ for the S_4_ Vertical Excited State of TMTQ in Nitromethane, Computed Using DFT and TD-DFT at the CAM-B3LYP/cc-pVDZ Level^a^

a Matrix elements for the *x* and *y* components of the **μ**_CT_ vector are omitted because their magnitudes are much smaller than those of the *z* component. Diagonal elements (highlighted in pink) correspond to *intra*-subdomain contributions, **μ**_CT,*z*_^intra,+^ and **μ**_CT,*z*_^intra,–^, to **μ**_CT,*z*_^+^ and **μ**_CT,*z*_^–^, respectively. Off-diagonal elements (highlighted in light blue) correspond to *inter-subdomain* contributions, **μ**_CT,*z*_^inter,+^ and **μ**_CT,*z*_^inter,–^, to **μ**_CT,*z*_^+^ and **μ**_CT,*z*_^–^, respectively (see eq 37).

The diagonal elements of the matrices (highlighted in pink) yield the *intra-subdomain* contributions, **μ**_CT,*z*_^intra,+^ and **μ**_CT,*z*_^intra,–^ (or **μ**_ST,*z*_^intra,↑^ and **μ**_ST,*z*_^intra,↓^) to **μ**_CT,*z*_^+^ and **μ**_CT,*z*_^–^ (or **μ**_ST,*z*_^↑^ and **μ**_ST,*z*_^↓^), respectively. The off-diagonal elements (highlighted in light blue) yield the *inter-subdomain* contributions **μ**_CT,*z*_^inter,+^ and **μ**_CT,*z*_^inter,–^ (or **μ**_ST,*z*_^inter,↑^ and **μ**_ST,*z*_^inter↓^).

The sign of each matrix element indicates whether the corresponding contribution concurs or opposes **μ**_CT,*z*_ or **μ**_ST,*z*_. For S_4_ ← S_0_ and T_5_ ← T_1_, **μ**_CT,*z*_ and **μ**_ST,*z*_ are negative and positive, respectively (Table S7 and Figures 8 and 9). Therefore, negative values in *M*_CT_^z,+^ and *M*_CT_^z,–^ correspond to contributions concurring with **μ**_CT,*z*_, whereas for *M*_ST_^z,↑^ and *M*_ST_^z,↓^ it is the positive elements that concur with **μ**_ST,*z*_.

The matrix elements in Tables 3 and S8 indicate which of the five subdomains is most effective in contributing to the intra-subdomain terms of **μ**_CT,*z*_^+^ , **μ**_CT,*z*_^–^, **μ**_ST,*z*_^↑^ , and **μ**_ST,*z*_^↓^, and which subdomains are more strongly coupled and therefore contribute most to the inter-subdomain terms.

Since the S_4_ ← S_0_ and T_5_ ← T_1_ excitations do not significantly break molecular symmetry, all matrices shown in Tables 3 and S8 reflect such symmetry and thus exhibit a clearly recognizable, nearly perfect 3-block structure (DCN = DCN1 ∪ DCN2; THIO = THIO1 ∪ THIO2; M10A).

The S_4_ ← S_0_ value of **μ**_CT,*z*_^+^ is predominantly (68%) determined by the *inter*-*subdomain* contributions (Table S7). Among these, the DCN groups contribute roughly twice as much as the THIO groups to the cross-terms with the M10A group, which almost entirely define **μ**_CT,*z*_^inter,+^ (−0.547 D out of −0.614 D). The opposite direction of the charge-excitation length in the THIO groups, which yields positive rather than negative matrix elements for **d**_CT,*z*_^THIO^ *q*_CT_^+^(M10A), accounts for their smaller contribution compared to the DCN groups. The intra-subdomain contribution arises almost exclusively from the M10A group (−0.293 D out of a total of −0.279 D).

In contrast to **μ**_CT,*z*_^+^ , the reconstruction of **μ**_CT,*z*_^–^ for the S_4_ ← S_0_ excitation is dominated (74%) by *intra*-*subdomain* contributions (Table S7), almost entirely due to the M10A group. Once again, the oppositely directed charge-transfer excitation length of the THIO groups, giving positive rather than negative values for **d**_CT,*z*_^THIO^ *q*_CT_^–^(M10A), results in their positive contribution (0.227 D) to the cross-terms with M10A, whereas the DCN groups contribute negatively (−0.430 D).

As for S_4_ ← S_0_, the **μ**_CT,*z*_^+^ value for T_5_ ← T_1_ (−4.755 D) is largely (77%) determined by *inter*-*subdomain* contributions (Table S7). As in S_4_ ← S_0_, the oppositely oriented charge-excitation length of the THIO groups causes the DCN groups to contribute about twice as much as the THIO groups to the cross-terms with the M10A group, which account for most of **μ**_CT,*z*_^inter,+^ (−2.667 D out of −3.657 D). The cross-terms involving DCN1-DCN2 and, especially, DCN-THIO pairs also contribute, yielding the remaining −0.990 D (including minor opposing contributions from the THIO1-THIO2 mixed terms). The intra-subdomain contribution arises mainly from the M10A and DCN groups (−0.678 and −0.479 D, respectively; total −1.098 D), slightly counterbalanced by the small opposing THIO contribution (+0.058 D).

The reconstruction of **μ**_CT,*z*_^–^ for T_5_ ← T_1_ is approximately equally partitioned between *inter*-*subdomain* and *intra*-*subdomain* contributions (Table S7). The latter (Table S8) is dominated by the M10A group. The *inter*-*subdomain* contribution arises mainly from the **d**_CT,*z*_^DCN^ *q*_CT_^–^(M10A) terms (−1.723 D), and to a much lesser extent from the **d**_CT,*z*_^M10A^ *q*_CT_^–^(THIO) terms (−0.425 D).

In contrast to **μ**_CT,*z*_^+^, the spin-transfer counterpart **μ**_ST,*z*_^↑^ for the T_5_ ← T_1_ excitation is about equally determined by *inter*-*subdomain* and *intra-subdomain* contributions. The former is essentially due to cross-terms involving the M10A group, contributing 2.551 D out of 2.938 D. The DCN-M10A cross-terms alone account for 2.522 D, whereas those involving the THIO excitation lengths oppose **μ**_ST,*z*_^↑^ and nearly cancel their own cross-terms contributions with M10A. The *intra*-*subdomain* component (54%) arises almost entirely from the M10A group.

The reconstruction of **μ**_ST,*z*_^↓^ is dominated by *inter*-*subdomain* contributions, mainly due to cross-terms with the M10A group, which total 3.669 D out of 4.681 D. Once again, the DCN groups contribute about twice as much as the THIO groups. The *intra*-*subdomain* contribution (1.704 D) arises from the M10A group (1.241 D) and the DCN groups (0.566 D), with a small opposing contribution from the THIO groups.

## Conclusions

We have presented a spin-polarized extension of our previously developed method for decomposing the Le Bahers, Adamo, Ciofini charge-transfer (CT) excitation global indexes^10^ into contributions from molecular subdomains. In addition to the global CT excitation indexes, we introduce their spin-transfer (ST) analogues and similarly decompose them into chemically meaningful subdomain contributions. This extension provides a coherent set of CT and ST descriptors, enabling both visual and quantitative differentiation of electronic charge- and spin-transfer behavior. The updated DOCTRINE_SPIN software now computes ST indexes and their descriptors, broadening the applicability of the method to spin-resolved electronic excitations.

The model enables a quantitative evaluation of subdomain contributions to CT, ST, their excitation lengths, and their charge- and spin-transfer dipole moments. Although these global indexes may be derived from either electron and spin density increments or from their depletions, the corresponding subdomain contributions generally differ. This distinction helps determine whether a subdomain’s contribution to a given property is dominated by one distribution or whether both play a substantial role, thus revealing the ambivalent nature of the subdomain.

As an application of our spin-polarized model extension, we studied a π-conjugated A–D–A compound (TMTQ), consisting of a central 1,6-methano[10]­annulene (M10A) and exo-positioned 5-dicyanomethyl-thiophene (DT) peripheries. TMTQ exhibits a small singlet–triplet energy gap of 4.9 kcal/mol, with the singlet state more stable than the triplet. This narrow energy gap stems from the different weights of nearly degenerate mesomeric structures with distinct electron delocalization patterns. Charge- and spin-density analyses of the singlet and triplet ground states enabled us to quantify these weights. We characterized and compared the electronic charge- and spin-transfer processes occurring upon excitation of S_0_ and T_1_ to their first five excited states, highlighting the distinctive features of each series, the role of ST in modulating CT when both processes are active, and the resulting effects on electron and spin delocalization. In the triplet states, spin polarization allows separations of the positive and negative CT poles that are an order of magnitude larger than in the corresponding singlet states. When CT and ST occur simultaneously, the charge dipole-moment change is markedly amplified due to the enhanced separation of the CT poles, even though the transferred charge is comparable to that in the singlet series. Spin-transfer dipole moments were found to be of similar magnitude and aligned but antiparallel to the charge-transfer dipole moments.

The atomic group decomposition of transferred charge shows that in four of the five singlet excitations, all selected subdomains behave ambivalently, with some regions contributing to the positive and others to the negative CT poles. In contrast, for the S_4_ ← S_0_ excitation—which exhibits by far the largest dipole-moment change and excitation length—the subdomains maximize their electron-withdrawing or electron-donating behavior, strongly reducing their ambivalence. This excitation corresponds to a state with the largest contribution from the Baird-aromatic (8π-electron)/polar structure. In the triplet series, the possibility of concurrent CT and ST leads to a much lower degree of ambivalence in the subdomains. For all excitations except T_6_ ← T_1_, the negative CT pole is dominated by the annulenic M10A subdomain.

As in the singlet series, the largest charge dipole-moment change and excitation length arise in the fourth excitation (T_5_), where subdomains again maximize their withdrawing/donating roles, and the strongest negative CT and largest decrease in M10A electron population are observed. Analogously, the largest ST dipole moment and spin excitation length also occur in the fourth excitation, where subdomains minimize spin ambivalence and both the strongest M10A spin-up transfer and the largest increase in M10A spin population take place.

For both singlet and triplet excitations, the largest contributions to the charge and spin-transfer dipole moments originate from mixed (inter-subdomain) terms, in which charge- or spin-transfer in one subdomain couples with the excitation-charge or spin-transfer length of another. Accordingly, intra-subdomain contributions to the charge- or spin-transfer dipole moments are generally less effective than those delocalized across two subdomains. Nevertheless, notable exceptions occur—particularly for excitations exhibiting the largest charge- or spin-transfer dipole moment within each series, and especially when considering decompositions based on charge- or spin-decrease contributions. Analysis of the subdomain charge- and spin-transfer dipole moment matrices provides further detailed insight into the individual and synergistic roles of the various subdomains in shaping the intra- and inter-subdomain contributions to the charge- and spin-transfer dipole moments.

The global CT and ST indexes encode underlying chemical motifs that would often be difficult to quantify—or even to hypothesize—without decomposition into suitable concurring or opposing contributions. The resulting subdomain contribution patterns offer a rich, chemically insightful representation of photoexcited transitions, whether spin-polarized or not. When spin polarization is active, the subdomain patterns for charge- and spin-related indexes provide a particularly clear picture of how spin and charge rearrangements jointly determine the observed values of these metrics.

Overall, our method offers a useful and distinctive tool among existing approaches for the study of light-driven charge- and spin-transfer processes.

## Supplementary Material



## Footnotes

a The values of Δ*q*_*CT*_(Ω) reported in Table 2 differ in sign and slightly in magnitude from those of Δ*q*_Ω_ shown in Table 1. These differences arise from the sign convention adopted for charge transfer, as well as from the use of a common subdomain boundary—specifically, that of the ground state—when evaluating the charge transfers.

## Notes

Calculated energies of geometry-optimized TMTQ in nitromethane for the singlet ground (S_0_) state and the biradical (T_0_) structure at the (U)­CAM-B3LYP/cc-pVDZ level using the SMD solvation model (Table S0–1); same as Table S0–1, but for geometry-optimized TMTQ *in vacuo* (Table S0–2); geometrical coordinates (Å) of the S_0_ and T_1_ states at the various computational levels adopted (Table S0–3); accuracy of the evaluation of the norm of the dipole moment change between ground and excited state *n*, and of the transferred charge (or of the transferred spin *s*_*ST*_), using the atomic group decomposition of the global CT indexes (Table S1); TMTQ *in vacuo* (DFT and TD-DFT at B3LYP-D3/6-311G­(d,p) level); Bader’s net charge and spin population changes in the singlet and triplet TMTQ molecule subdomains upon excitation, along with the corresponding changes in the values of descriptors related to electron delocalization in the annulenic ring (Table S2); TMTQ *in vacuo* (DFT and TD-DFT at CAM-B3LYP-D3­(BJ)/cc-pVDZ level). Bader’s net charge and spin population changes in the singlet and triplet TMTQ molecule subdomains upon excitation, along with the corresponding changes in the values of descriptors related to electron delocalization in the annulenic ring (Table S3); TMTQ *in vacuo* (DFT and TD-DFT at CAM-B3LYP/cc-pVDZ level). Bader’s net charge and spin population changes in the singlet and triplet TMTQ molecule subdomains upon excitation, along with the corresponding changes in the values of descriptors related to electron delocalization in the annulenic ring (Table S4); atomic group decomposition of the norms of the charge- and spin-transfer dipole moments, for the first five singlet and triplet vertical excited states of TMTQ in nitromethane (Table S5); atomic group decomposition of the charge-transfer (CT) and spin-transfer (ST) excitation lengths, *D*_*CT*_ and *D*_*ST*_, for the first five singlet and triplet vertical excited states of TMTQ in nitromethane (Table S6); decomposition of the *z*-component of the charge-transfer (CT) and spin-transfer (ST) dipole moment vectors, into *intra*-subdomain and *inter*-subdomain contributions (Table S7); and matrix elements (in Debye, D) of *M*_*CT*_^*z*,+^ and *M*_*CT*_^*z*,–^ (or *M*_*ST*_^*z*,↑^ and *M*_*ST*_^*z*,↓^) for the T_5_ vertical excited state of TMTQ in nitromethane (Table S8) (PDF)

## Notes

This paper is dedicated to Francesc Illas and Gianfranco Pacchioni.
