# Supplementary material for: Decomposition of Molecular Charge and Spin Transfer Global Indexes into Atomic Group Contributions
Source: J Chem Theory Comput. 2026 Apr 3;22(8):4049–70. doi: 10.1021/acs.jctc.5c02145 (PMC13130860; doi:10.1021/acs.jctc.5c02145)
Supplement: Supplementary file 1 [file ct5c02145_si_001.pdf]

Supplementary Information (S0-S8)

Decomposition of Molecular Charge and Spin Transfer Global Indexes into Atomic Group Contributions

Carlo Gatti *[a,b], Yann Danten [c] and Christine Frayret [d]

a) CNR SCITEC, CNR Istituto di Scienze e Tecnologie Chimiche ''Giulio Natta'', Sede Via C. Golgi, 19, 20133 Milano, Italy

b) Istituto Lombardo, Accademia di Scienze e Lettere, via Brera 76, 20133 Milano, Italy

c) Institut des Sciences Moléculaires, UMR CNRS 5255, 351 Cours de la Libération, 33405 Talence, France

d) Laboratoire de Réactivité et Chimie des Solides (LRCS), UMR CNRS 7314, Université de Picardie Jules Verne, Hub de l'Energie, 15 Rue Baudelocque, 80000 Amiens Cedex, France. Réseau sur le Stockage Electrochimique de l'Energie (RS2E), FR CNRS 3459, France.

(*) Orcid 0000-0002-0047-1596; e-mail c.gatti@scitec.cnr.it

Table S0-1. Calculated energies of geometry-optimized TMTQ in nitromethane for the singlet ground (S_0) state and the biradical (T_0) structure at the (U)CAM-B3LYP/cc-pVDZ level using the SMD solvation model. The electronic energy (E°), zero-point energy–corrected electronic energy (E° + ZPE), internal energy, enthalpy, and Gibbs free energy at 298 K are also reported in Hartree units.

TMTQ	E° (H)	E^{o+ZPE} (H)	U^{298K} (H)	H^{298K} (H)	G^{298K} (H)	μ (D)
**Ground State S0**	-1974.56146	-1974.26064	-1974.23449	-1974.23355	-1974.31924	4.80
**Bi-radical S=1**	-1974.55217	-1974.25351	-1974.22723	-1974.22629	-1974.31442	9.93

Table S0-2. Same as Table S0-1, but for geometry-optimized TMTQ *in vacuo*. Energies are reported for the singlet ground (S_0) state and the biradical (T_0) structure, computed at the (U)B3LYP-D3(BJ)/6-311G(d,p) and (U)CAM-B3LYP-D3(BJ)/cc-pVDZ levels, respectively.

TMTQ	E° (H)	E^{o+ZPE} (H)	U^{298K} (H)	H^{298K} (H)	G^{298K} (H)	μ (D)
**B3LYP-D3(BJ)/6-311G(d,p)**						
**Ground State S0**	-1975.54167	-1975.24507	-1975.21868	-1975.21774	-1975.30399	3.735
**Bi-radical S=1**	-1975.53382	-1975.23870	-1975.21229	-1975.21134	-1975.29929	6.811
**CAM-B3LYP-D3(BJ)/cc-pVDZ**						
**Ground State S0**	-1974.58638	-1974.28495	-1974.25875	-1974.25780	-1974.34368	2.736
**Bi-radical S=1**	-1974.58337	-1974.28465	-1974.25838	-1974.25744	-1974.34529	6.739

Table S-03 Coordinates (Angstrom)

(U)CAM-B3LYP/cc-pVDZ with SMD(Nitromethane)

(S0)

C	-1.03684700	0.52536300	0.67373700
C	-0.76662100	1.62811200	-0.06265300
C	-2.13369200	-0.41189200	0.37818500
C	0.00070900	-0.00185900	1.61794800
C	0.51104500	2.32258100	-0.03685300
H	-1.50415500	2.00312500	-0.77626800
C	-1.75533600	-1.80475500	0.16052700
C	-3.43482800	0.00371400	0.28127900
C	1.03716700	-0.52332700	0.66938800
H	-0.40362100	-0.80108600	2.25044200
H	0.40600200	0.79369100	2.25444400
C	1.75503600	1.81013500	0.16991800
H	0.47431500	3.38262600	-0.30434000
C	-0.51170900	-2.31590500	-0.05123100
H	-2.58075400	-2.51750000	0.08620900
C	-3.97296600	1.29636300	0.60892200
S	-4.75696900	-1.05088000	-0.27828600
C	2.13353100	0.41586000	0.37759000
C	0.76607100	-1.62162000	-0.07343400
H	2.58036400	2.52336800	0.10100000
H	-0.47555000	-3.37378000	-0.32715500
C	-5.30970400	1.42181200	0.41744900
H	-3.34196500	2.09032700	1.00426800
C	-5.93225400	0.22443500	-0.07630500
C	3.43419700	-0.00062100	0.27632900
H	1.50252400	-1.99215700	-0.79036300
H	-5.88557600	2.32152900	0.62743100
C	-7.27430400	0.05268900	-0.36230700
C	3.96975300	-1.29385600	0.60483100
S	4.75813700	1.05086400	-0.28498800
C	-8.19568400	1.12638600	-0.19927100
C	-7.78065900	-1.19305300	-0.82814600
C	5.30657400	-1.42137700	0.41595200
H	3.33647400	-2.08558700	1.00089900
C	5.93163000	-0.22598800	-0.07916300
N	-8.94021300	2.00529200	-0.06381700
N	-8.16912500	-2.21846300	-1.20677700
H	5.88045900	-2.32141900	0.62930300
C	7.27472000	-0.05692400	-0.36242100
C	7.78343600	1.18518400	-0.83525900
C	8.19521200	-1.12958300	-0.18759900
N	8.17426100	2.20739100	-1.22009300
N	8.93878300	-2.00766500	-0.04188000

(T0)

C	-1.32963900	0.54145400	1.13085100
C	-0.84847500	1.81806900	0.79955300
C	-2.26954800	-0.23334500	0.38352400
C	-0.68557000	-0.14168400	2.28458300
C	0.41316800	2.27000200	1.17353700
H	-1.43654100	2.46778800	0.14889900
C	-2.09828200	-1.62259700	0.28229500
C	-3.36440600	0.41054300	-0.32901700
C	0.50525800	-0.71171100	1.59926300
H	-1.32647200	-0.92756000	2.69848500
H	-0.40099300	0.57025800	3.06672800
C	1.54007000	1.50672000	1.55414600
H	0.62749700	3.32386400	0.97833500
C	-0.92196000	-2.37532200	0.49433600
H	-2.91083300	-2.18044700	-0.19049500
C	-4.02667200	1.59274000	0.00043600
S	-4.05343800	-0.30143800	-1.77098700
C	1.67529600	0.11066900	1.57939300
C	0.32529900	-1.92457900	0.91771400
H	2.45973800	2.08706200	1.66512100
H	-0.96062700	-3.39381100	0.09958600
C	-5.06478400	1.91964200	-0.86462000
H	-3.76624900	2.18166900	0.87829600
C	-5.23384000	0.98449900	-1.90531300
C	3.00178600	-0.48584300	1.52031200
H	1.19433200	-2.51422400	0.62024200
H	-5.70464900	2.79448500	-0.75657300
C	-6.18643000	1.02462600	-2.93291900
C	3.40958200	-1.72542100	2.01326200
S	4.35124400	0.36651800	0.80268200
C	-7.11604800	2.09655800	-3.01662200
C	-6.26066800	0.00961200	-3.92361000
C	4.76292800	-1.98777100	1.83540000
H	2.72615800	-2.40507300	2.51947400
C	5.44973000	-0.94099900	1.18783200
N	-7.87095700	2.97548700	-3.07595200
N	-6.30470900	-0.83029300	-4.72330200
H	5.26472900	-2.89421200	2.17171300
C	6.81301100	-0.89488000	0.86606000
C	7.39183700	0.23193400	0.22367300
C	7.66667400	-1.98889600	1.17414100
N	7.84565900	1.16215100	-0.30133800
N	8.35559400	-2.88665800	1.42970900

(U)B3LYP-D3(BJ)/6-311G(d,p)

(S0)

C	-1.04250600	0.51619500	0.77221000
C	-0.78300900	1.64818400	0.06339200
C	-2.11631200	-0.43098100	0.46728900
C	0.00007700	-0.00003100	1.71832900
C	0.48485100	2.33255200	0.07269700
H	-1.53156000	2.02890900	-0.62348400
C	-1.73651100	-1.81258400	0.27490000
C	-3.42560000	-0.01422300	0.32219700
C	1.04250300	-0.51619200	0.77200200
H	-0.39808800	-0.79691900	2.34587900
H	0.39834000	0.79683200	2.34584700
C	1.73646100	1.81260100	0.27472800
H	0.45611800	3.37734600	-0.22284100
C	-0.48493500	-2.33252000	0.07263900
H	-2.55941300	-2.51416700	0.18276400
C	-3.97359000	1.25924700	0.67745500
S	-4.70699000	-1.05209400	-0.33748800
C	2.11628800	0.43099900	0.46704400
C	0.78290800	-1.64812200	0.06314000
H	2.55935000	2.51418600	0.18249000
H	-0.45623800	-3.37730400	-0.22293900
C	-5.30715700	1.38836500	0.44120800
H	-3.36120100	2.02562600	1.13044100
C	-5.90595100	0.21360100	-0.12061100
C	3.42557700	0.01424100	0.32197300
H	1.53136500	-2.02879100	-0.62386900
H	-5.89706300	2.26556600	0.66720300
C	-7.23502900	0.04823300	-0.45790000
C	3.97353200	-1.25922700	0.67728300
S	4.70701500	1.05209600	-0.33764100
C	-8.15151200	1.11716900	-0.28025900
C	-7.71049500	-1.17943800	-0.98370700
C	5.30711300	-1.38836000	0.44113100
H	3.36110300	-2.02557900	1.13025600
C	5.90595500	-0.21360600	-0.12066200
N	-8.87270400	2.00757100	-0.12209800
N	-8.05878200	-2.19711800	-1.40995700
H	5.89699800	-2.26555800	0.66718600
C	7.23506000	-0.04825700	-0.45784900
C	7.71059000	1.17940300	-0.98362200
C	8.15151900	-1.11719800	-0.28010400
N	8.05893300	2.19707600	-1.40984300
N	8.87268700	-2.00760300	-0.12185500

(T0)

C	-1.03978000	0.48760900	1.41314600
C	-0.85052400	1.75861700	0.86314700
C	-2.02319300	-0.47011500	1.01660300
C	0.00003800	0.00004600	2.36562300
C	0.38936500	2.39958100	0.83326200
H	-1.66901300	2.23907900	0.34015500
C	-1.67380400	-1.83119000	0.93932100
C	-3.34619900	-0.03936800	0.61978200
C	1.03972100	-0.48757300	1.41302300
H	-0.37915300	-0.80863700	2.98805900
H	0.37930900	0.80875800	2.98797200
C	1.67370200	1.83119600	0.93895300
H	0.38300200	3.43678400	0.51351300
C	-0.38951000	-2.39957700	0.83351200
H	-2.49171400	-2.52014000	0.75515900
C	-4.04436400	1.08752100	1.05667800
S	-4.31040800	-0.96420300	-0.51424600
C	2.02313600	0.47016400	1.01634800
C	0.85039100	-1.75855600	0.86310000
H	2.49156900	2.52015900	0.75465000
H	-0.38317700	-3.43681600	0.51387500
C	-5.31946500	1.20939100	0.51512200
H	-3.62998500	1.77250600	1.78285900
C	-5.64725400	0.16416100	-0.36744500
C	3.34614700	0.03940400	0.61961700
H	1.66876900	-2.23900000	0.33991700
H	-6.01665900	2.00221800	0.74807200
C	-6.85235400	-0.00864800	-1.06998300
C	4.04421500	-1.08757200	1.05646700
S	4.31051600	0.96428400	-0.51424900
C	-7.88780900	0.94508800	-0.93715400
C	-7.05832000	-1.12252500	-1.91355400
C	5.31934600	-1.20947400	0.51500700
H	3.62974100	-1.77260200	1.78255000
C	5.64727400	-0.16418600	-0.36744600
N	-8.71505500	1.74458500	-0.80493700
N	-7.19324900	-2.04864900	-2.59640100
H	6.01646900	-2.00236500	0.74795000
C	6.85243500	0.00860700	-1.06987500
C	7.05854400	1.12256100	-1.91330800
C	7.88780500	-0.94522800	-0.93708600
N	7.19359100	2.04874900	-2.59604500
N	8.71497800	-1.74480500	-0.80489900

(U)CAM-B3LYP-D3(BJ)/cc-pVDZ

(S0)

C	-1.03841300	0.52584600	0.61063100
C	-0.76621800	1.62775400	-0.12331900
C	-2.13362800	-0.41612600	0.32707000
C	0.00004500	-0.00001400	1.55618200
C	0.51158800	2.32453900	-0.09735900
H	-1.50138500	1.99058100	-0.84570400
C	-1.75253600	-1.80912800	0.11071500
C	-3.43440800	-0.00811100	0.24548200
C	1.03841400	-0.52586800	0.61052700
H	-0.40805200	-0.79720500	2.18892600
H	0.40820000	0.79718100	2.18888300
C	1.75250600	1.80910300	0.11057000
H	0.47755600	3.38443400	-0.36508400
C	-0.51163700	-2.32456500	-0.09731900
H	-2.58297200	-2.51606000	0.04141000
C	-3.96307700	1.29378200	0.58100900
S	-4.76190700	-1.06344400	-0.28805900
C	2.13361500	0.41610600	0.32691900
C	0.76616000	-1.62776900	-0.12340500
H	2.58293600	2.51603500	0.04118200
H	-0.47762500	-3.38446300	-0.36503200
C	-5.29843100	1.41944900	0.41882200
H	-3.31637100	2.08184300	0.96135300
C	-5.93740800	0.21521000	-0.06144900
C	3.43439900	0.00809900	0.24534500
H	1.50126800	-1.99058400	-0.84585500
H	-5.87755400	2.31470000	0.63816200
C	-7.27561800	0.05457500	-0.31140400
C	3.96306300	-1.29379900	0.58086700
S	4.76191200	1.06344700	-0.28813400
C	-8.17393300	1.14829600	-0.11762200
C	-7.79815100	-1.19336800	-0.76583500
C	5.29842200	-1.41945600	0.41872200
H	3.31634500	-2.08186600	0.96117700
C	5.93741000	-0.21520400	-0.06149800
N	-8.87286400	2.05671600	0.05134000
N	-8.18404500	-2.22202500	-1.13376800
H	5.87754200	-2.31470700	0.63806700
C	7.27563400	-0.05455400	-0.31137300
C	7.79819100	1.19340400	-0.76573400
C	8.17394300	-1.14827500	-0.11756200
N	8.18410500	2.22207400	-1.13360900
N	8.87286900	-2.05669400	0.05142400

(T0)

C	-1.03132000	0.47577800	1.48866300
C	-0.86178700	1.76586700	0.96387100
C	-2.00303700	-0.48503800	1.08302800
C	-0.00000900	-0.00021000	2.44862200
C	0.36897500	2.41202600	0.93585600
H	-1.69749200	2.25771900	0.46393000
C	-1.65924700	-1.84118800	1.02113600
C	-3.33059200	-0.05518000	0.65850700
C	1.03132800	-0.47605100	1.48861600
H	-0.37725100	-0.81813300	3.07182000
H	0.37721500	0.81761600	3.07195600
C	1.65926300	1.84099600	1.02149800
H	0.35884600	3.45902200	0.62303100
C	-0.36894900	-2.41221100	0.93545300
H	-2.47912300	-2.53486000	0.81892600
C	-4.05863600	1.03613500	1.11958300
S	-4.23209000	-0.94478100	-0.54049000
C	2.00305000	0.48482600	1.08315900
C	0.86181400	-1.76607200	0.96363100
H	2.47914500	2.53470500	0.81944400
H	-0.35881400	-3.45915500	0.62245300
C	-5.32034000	1.15814200	0.53669100
H	-3.67905300	1.70450700	1.89042100
C	-5.59194600	0.14547200	-0.39905900
C	3.33060600	0.05505100	0.65856000
H	1.69754300	-2.25786200	0.46367100
H	-6.04538900	1.93428100	0.77675500
C	-6.76909000	-0.01760200	-1.14873700
C	4.05866300	-1.03634300	1.11942500
S	4.23208700	0.94487900	-0.54028400
C	-7.83234800	0.91426500	-1.00464900
C	-6.92751900	-1.09836200	-2.05547500
C	5.32036100	-1.15823600	0.53649600
H	3.67909500	-1.70486500	1.89014200
C	5.59194900	-0.14539400	-0.39907300
N	-8.67933900	1.69409900	-0.86059300
N	-7.02596600	-1.99338700	-2.78780300
H	6.04541700	-1.93441400	0.77641200
C	6.76907500	0.01782200	-1.14875000
C	6.92746500	1.09873900	-2.05530800
C	7.83234600	-0.91405900	-1.00485700
N	7.02587900	1.99389000	-2.78748600
N	8.67935000	-1.69390800	-0.86096100

Table S1. TMTQ in nitromethane (DFT and TD-DFT at CAM-B3LYP/cc-pVDZ level). Accuracy of the evaluation of the norm of the dipole moment change between the ground and the excited state *n,* $||\boldsymbol{\mu}_{CT}||$, and of the transferred charge q_{CT} (or of the transferred spin s_{ST}), upon excitation, using the atomic group subdomains decomposition of the global CT indexes, for all the investigated BM excitations

State	$\|\|\boldsymbol{\mu}_{CT}\|\|, D$	$\Delta\|\|\boldsymbol{\mu}_{CT}\|\|, D$ [a]	q_{CT}^{+} [b]	q_{CT}^{-} [b]	$q_{CT}^{+} - q_{CT}^{-}$	$s_{ST}^{\uparrow}$ [b]	$s_{ST}^{\downarrow}$ [b]	$s_{ST}^{\uparrow} - s_{ST}^{\downarrow}$
**Singlet states values**								
$S_1 \leftarrow S_0$	0.0503	-0.0002	0.4963	0.4963	$7.\cdot 10^{-6}$	-	-	-
$S_2 \leftarrow S_0$	0.1824	+0.0007	0.3844	0.3844	$9.\cdot 10^{-6}$	-	-	-
$S_3 \leftarrow S_0$	0.3490	+0.0003	0.4818	0.4818	$1.\cdot 10^{-5}$	-	-	-
$S_4 \leftarrow S_0$	0.8957	-0.0025	0.7928	0.7925	0.0003	-	-	-
$S_5 \leftarrow S_0$	0.0940	+0.0006	0.4504	0.4504	$1.\cdot 10^{-6}$	-	-	-
**Triplet states: charge (*and spin*) values**								
$T_2 \leftarrow T_1$	1.2499	-0.0015	0.5003	0.5003	$-2.\cdot 10^{-5}$	0.9500	0.9500	$6.\cdot 10^{-6}$
$T_3 \leftarrow T_1$	2.2692	-0.0016	0.5528	0.5527	0.0001	1.0696	1.0696	$-4.\cdot 10^{-5}$
$T_4 \leftarrow T_1$	1.7675	-0.0014	0.4014	0.4015	$5.\cdot 10^{-6}$	0.6606	0.6606	$-8.\cdot 10^{-5}$
$T_5 \leftarrow T_1$	4.7588	-0.0021	0.8309	0.8307	0.0002	1.2736	1.2736	$2.\cdot 10^{-5}$
$T_6 \leftarrow T_1$	1.5159	-0.0002	0.4742	0.4742	$-2.\cdot 10^{-5}$	1.0706	1.0706	$-2.\cdot 10^{-5}$

a) Dipole moments are reported in Debye (D). $\Delta||\boldsymbol{\mu}_{CT}|| = ||\boldsymbol{\mu}_{CT}||_{\text{DOCTRINE\_SPIN}} - ||\boldsymbol{\mu}_{CT}||_{\text{Gaussian 16}}$ represents the difference (shown in column 2 of the Table) between the $||\boldsymbol{\mu}_{CT}||$ value obtained from the individual atomic or atomic group subdomain contributions, as evaluated by the DOCTRINE_SPIN code, and the $||\boldsymbol{\mu}_{CT}||$ value derived from the dipole moment components of the two electronic states involved, as calculated using the Gaussian-16 code.

b) q_{CT}^{+} and q_{CT}^{-} ($s_{ST}^{\uparrow}$ and $s_{ST}^{\downarrow}$) are the values, upon excitation, of the transferred charge q_{CT} (or of the transferred spin s_{ST}), calculated by integrating $\rho^{+}(\boldsymbol{r})$ or $\rho^{-}(\boldsymbol{r})$, ($s^{+}(\boldsymbol{r})$ or $s^{-}(\boldsymbol{r})$) over the entire set of atomic or atomic group subdomains of the TMTQ molecule.

Table S2. TMTQ *in vacuo* (DFT and TD-DFT at B3LYP-D3/6-311G(d,p) level). Bader's Net Charge ($\Delta q_{CT,\Omega}$) and spin population ($\Delta s_{CT,\Omega}$) changes in the singlet and triplet TMTQ molecule subdomains Ω upon excitation, along with the corresponding changes in the values of descriptors related to electron delocalization in the annulenic ring (M10A). Net charges, spin populations and their changes upon excitation are reported, in e^-, for the molecular subdomains: DCN = DCN1∪DCN2, THIO = THIO1∪THIO2 and M10A. For the reference S_0 and T_1 states, the net charges and spin populations, in e^-, of the M10A, THIO, and DCN subdomains are reported, together with geometrical and electronic descriptors related to electron delocalization in the M10A annulenic ring. All data for excitations between triplet states in *italic*

Excitation	Δq_{M10A}	Δs_{M10A}	Δq_{THIO}	Δs_{THIO}	Δq_{DCN}	Δs_{DCN}	ΔHOMA[a]	ΔFLU[a]	Δ*std*$_R$[a]	Δ*std*$_{DI}$[a]
$S_1 \leftarrow S_0$	0.006	-	-0.015	-	0.010	-	-	-0.007	-	-0.042
$T_2 \leftarrow T_1$	*0.083*	*0.259*	*-0.044*	*-0.083*	*-0.040*	*-0.176*	-	*0.005*	-	*0.022*
$S_2 \leftarrow S_0$	-0.017	-	-0.018	-	0.043	-	-	-0.005	-	-0.028
$T_3 \leftarrow T_1$	*0.119*	*0.394*	*-0.071*	*-0.101*	*-0.048*	*-0.293*	-	*0.001*	-	*0.001*
$S_3 \leftarrow S_0$	-0.059	-	0.027	-	0.039	-	-	-0.007	-	-0.044
$T_4 \leftarrow T_1$	*0.120*	*0.456*	*-0.053*	*-0.213*	*-0.068*	*-0.243*	-	*0.004*	-	*0.012*
$S_4 \leftarrow S_0$	0.026	-	-0.024	-	0.005	-	-	-0.007	-	-0.044
$T_5 \leftarrow T_1$	*0.166*	*0.474*	*-0.079*	*-0.229*	*-0.088*	*-0.245*	-	*0.003*	-	*-0.000*
$S_5 \leftarrow S_0$	0.179	-	-0.114	-	-0.073	-	-	-0.011	-	-0.077
$T_6 \leftarrow T_1$	*0.100*	*0.241*	*-0.029*	*0.029*	*-0.071*	*-0.269*	-	*0.003*	-	*0.012*
$T_1 \leftarrow S_0$	-0.059	0.257	-0.020	0.684	0.078	1.059	0.533	-0.020	-0.030	-0.136
State	q_{M10A}	s_{M10A}	q_{THIO}	s_{THIO}	q_{DCN}	s_{DCN}	HOMA[a]	FLU[a]	*std*$_R$[a]	*std*$_{DI}$[a]
S_0	0.302	-	0.283	-	-0.574	-	0.332	0.025	0.042	0.199
T_1	0.243	0.257	0.263	0.684	-0.496	1.059	0.865	0.005	0.012	0.063

a) HOMA and FLU are geometric and electronic descriptors of electronic delocalization (see text). *std*$_R$, in Å, and *std*$_{DI}$ are the standard deviations of the C-C bond distances and of the delocalization indexes. The changes in all these quantities Z upon excitation (final - initial state) are denoted as ΔZ (last 4 columns for state excitation changes). For vertical excitations, the changes in the geometrical descriptors are zero by definition and are not reported. Geometric and electronic descriptors are evaluated using only data of the annulenic 10-CMR (10-Carbon Membered Ring) bonds, *i.e.* the data for bonds including the apical C atom are not included

Table S3. TMTQ *in vacuo* (DFT and TD-DFT at CAM-B3LYP-D3(BJ)/cc-pVDZ level). Bader's Net Charge ($\Delta q_{CT,\Omega}$) and spin population ($\Delta s_{CT,\Omega}$) changes in the singlet and triplet TMTQ molecule subdomains Ω upon excitation, along with the corresponding changes in the values of descriptors related to electron delocalization in the annulenic ring (M10A). Net charges, spin populations and their changes upon excitation are reported, in e^-, for the molecular subdomains: DCN = DCN1∪DCN2, THIO = THIO1∪THIO2 and M10A. For the reference S_0 and T_1 states, the net charges and spin populations, in e^-, of the M10A, THIO, and DCN subdomains are reported, together with geometrical and electronic descriptors related to electron delocalization in the M10A annulenic ring. All data for excitations between triplet states in *italic*

Excitation	Δq_{M10A}	Δs_{M10A}	Δq_{THIO}	Δs_{THIO}	Δq_{DCN}	Δs_{DCN}	ΔHOMA[a]	ΔFLU[a]	Δ*std*R[a]	Δ*std*DI[a]
$S_1 \leftarrow S_0$	0.028	-	-0.022	-	-0.005	-	-	-0.016	-	-0.072
$T_2 \leftarrow T_1$	*0.045*	*0.065*	*-0.026*	*-0.015*	*-0.016*	*-0.050*	-	*0.011*	-	*0.005*
$S_2 \leftarrow S_0$	0.015	-	-0.024	-	0.007	-	-	-0.011	-	-0.052
$T_3 \leftarrow T_1$	*0.078*	*0.163*	*-0.025*	*0.152*	*-0.054*	*-0.315*	-	*0.002*	-	*-0.004*
$S_3 \leftarrow S_0$	-0.030	-	0.002	-	0.024	-	-	-0.011	-	-0.054
$T_4 \leftarrow T_1$	*0.077*	*0.195*	*-0.005*	*0.052*	*-0.074*	*-0.247*	-	*0.004*	-	*-0.005*
$S_4 \leftarrow S_0$	-0.004	-	0.020	-	-0.014	-	-	-0.010	-	-0.048
$T_5 \leftarrow T_1$	*0.166*	*0.459*	*-0.066*	*-0.213*	*-0.100*	*-0.246*	-	*0.003*	-	*-0.002*
$S_5 \leftarrow S_0$	0.084	-	-0.051	-	-0.043	-	-	-0.019	-	-0.123
$T_6 \leftarrow T_1$	*0.026*	*0.059*	*0.061*	*0.288*	*-0.087*	*-0.348*	-	*0.001*	-	*-0.005*
$T_1 \leftarrow S_0$	-0.074	0.175	-0.030	0.657	0.091	1.168	0.854	-0.034	-0.041	-0.186
**State**	q_{M10A}	s_{M10A}	q_{THIO}	s_{THIO}	q_{DCN}	s_{DCN}	HOMA[a]	FLU[a]	*std*R[a]	*std*DI[a]
S_0	0.283	-	0.295	-	-0.561	-	0.020	0.038	0.053	0.254
T_1	0.209	0.175	0.266	0.657	-0.470	1.168	0.874	0.004	0.012	0.068

a) HOMA and FLU are geometric and electronic descriptors of electronic delocalization (see text). std_R, in Å, and std_{DI} are the standard deviations of the C-C bond distances and of the delocalization indexes. The changes in all these quantities *Z* upon excitation (final - initial state) are denoted as Δ*Z* (last 4 columns for state excitation changes). For vertical excitations, the changes in the geometrical descriptors are zero by definition and are not reported. Geometric and electronic descriptors are evaluated using only data of the annulenic 10-CMR (10-Carbon Membered Ring) bonds, *i.e.* the data for bonds including the apical C atom are not included

Table S4. TMTQ *in vacuo* (DFT and TD-DFT at CAM-B3LYP/cc-pVDZ level). Bader's Net Charge ($\Delta q_{CT,\Omega}$) and spin population ($\Delta s_{CT,\Omega}$) changes in the singlet and triplet TMTQ molecule subdomains Ω upon excitation, along with the corresponding changes in the values of descriptors related to electron delocalization in the annulenic ring (M10A). Net charges, spin populations and their changes upon excitation are reported, in e^-, for the molecular subdomains: DCN = DCN1∪DCN2, THIO = THIO1∪THIO2 and M10A. For the reference S_0 and T_1 states, the net charges and spin populations, in e^-, of the M10A, THIO, and DCN subdomains are reported, together with geometrical and electronic descriptors related to electron delocalization in the M10A annulenic ring. All data for excitations between triplet states in *italic*

Excitation	Δq_{M10A}	Δs_{M10A}	Δq_{THIO}	Δs_{THIO}	Δq_{DCN}	Δs_{DCN}	ΔHOMA[a]	ΔFLU[a]	Δstd_R[a]	Δstd_{DI}[a]
$S_1 \leftarrow S_0$	0.028	-	-0.024	-	-0.002	-	-	-0.016	-	-0.072
$T_2 \leftarrow T_1$	*0.042*	*0.063*	*-0.027*	*-0.014*	*-0.014*	*-0.048*	-	*0.010*	-	*0.006*
$S_2 \leftarrow S_0$	0.017	-	-0.027	-	0.010	-	-	-0.011	-	-0.052
$T_3 \leftarrow T_1$	*0.078*	*0.161*	*-0.024*	*0.154*	*-0.054*	*-0.315*	-	*0.002*	-	*-0.004*
$S_3 \leftarrow S_0$	-0.025	-	-0.000	-	0.028	-	-	-0.011	-	-0.054
$T_4 \leftarrow T_1$	*0.077*	*0.196*	*-0.003*	*0.052*	*-0.074*	*-0.248*	-	*0.004*	-	*-0.004*
$S_4 \leftarrow S_0$	-0.002	-	0.020	-	-0.016	-	-	-0.010	-	-0.049
$T_5 \leftarrow T_1$	*0.168*	*0.459*	*-0.068*	*-0.212*	*-0.100*	*-0.247*	-	*0.003*	-	*-0.002*
$S_5 \leftarrow S_0$	0.110	-	-0.062	-	-0.046	-	-	-0.019	-	-0.122
$T_6 \leftarrow T_1$	*0.024*	*0.056*	*0.066*	*0.294*	*-0.090*	*-0.351*	-	*0.001*	-	*-0.004*
$T_1 \leftarrow S_0$	-0.062	0.175	-0.031	0.654	0.087	1.171	0.871	-0.033	-0.041	-0.187
State	q_{M10A}	s_{M10A}	q_{THIO}	s_{THIO}	q_{DCN}	s_{DCN}	HOMA[a]	FLU[a]	std_R[a]	std_{DI}[a]
S_0	0.273	-	0.294	-	-0.558	-	-0.007	0.038	0.053	0.255
T_1	0.212	0.175	0.264	0.654	-0.471	1.171	0.864	0.005	0.012	0.068

a) HOMA and FLU are geometric and electronic descriptors of electronic delocalization (see text). std_R, in Å, and std_{DI} are the standard deviations of the C-C bond distances and of the delocalization indexes. The changes in all these quantities Z upon excitation (final - initial state) are denoted as ΔZ (last 4 columns for state excitation changes). For vertical excitations, the changes in the geometrical descriptors are zero by definition and are not reported. Geometric and electronic descriptors are evaluated using only data of the annulenic 10-CMR (10-carbon membered ring) bonds, *i.e.* the data for bonds including the apical C atom are not included

Table S5. Atomic group decomposition of the norms of the charge- and spin-transfer dipole moments, $||\boldsymbol{\mu}_{CT}||$ and $||\boldsymbol{\mu}_{ST}||$ (Debye, D), for the first five singlet and triplet vertical excited states of TMTQ in nitromethane, computed at DFT and TD-DFT CAM-B3LYP/cc-pVDZ level. The table reports $||\boldsymbol{\mu}^{+}_{CT}(\Omega)||$ and $||\boldsymbol{\mu}^{-}_{CT}(\Omega)||$ (and $\boldsymbol{\mu}^{\uparrow}_{ST}(\Omega)$ and $\boldsymbol{\mu}^{\downarrow}_{ST}(\Omega)$ for spin) contributing to $||\boldsymbol{\mu}_{CT}||$ *and* $||\boldsymbol{\mu}_{ST}||$, obtained according to Eqs. 27a-b (charge) and 27c-d (spin). These expressions retain only the dependence of $||\boldsymbol{\mu}_{CT}||$ on q_{CT} and of $||\boldsymbol{\mu}_{ST}||$ on s_{ST}. For each excitation, the largest $\boldsymbol{\mu}^{+}_{CT}(\Omega)$ and $\boldsymbol{\mu}^{-}_{CT}(\Omega)$ values (or $\boldsymbol{\mu}^{\uparrow}_{ST}(\Omega)$ and $\boldsymbol{\mu}^{\downarrow}_{ST}(\Omega)$) are highlighted in bold. All data were obtained using the DOCTRINE_SPIN code.

State	$\|\mu_{CT}\|$,<br>$\|\mu_{ST}\|$	$\mu^{+}_{CT}(\Omega)$, $\mu^{\uparrow}_{ST}(\Omega)$			$\mu^{-}_{CT}(\Omega)$, $\mu^{\downarrow}_{ST}(\Omega)$		
		DCN	THIO	M10A	DCN	THIO	M10A
**Singlet states**							
$S_1 \leftarrow S_0$	0.050	0.004	0.022	**0.024**	0.004	0.018	**0.029**
$S_2 \leftarrow S_0$	0.182	0.022	**0.111**	0.050	0.032	**0.088**	0.062
$S_3 \leftarrow S_0$	0.349	0.026	**0.176**	0.146	0.066	**0.144**	0.138
$S_4 \leftarrow S_0$	0.896	0.229	**0.343**	0.324	0.034	0.112	**0.748**
$S_5 \leftarrow S_0$	0.094	0.012	**0.049**	0.033	0.012	0.034	**0.046**
**Triplet states: charge (*and spin*) values**							
$T_2 \leftarrow T_1$	1.250<br>*1.225*	0.278<br>*0.053*	0.408<br>*0.473*	**0.564**<br>***0.699***	0.054<br>*0.216*	0.211<br>***0.550***	**0.984**<br>*0.457*
$T_3 \leftarrow T_1$	2.269<br>*2.947*	0.887<br>*0.277*	**1.108**<br>***1.609***	0.274<br>*1.061*	0.143<br>*1.102*	0.399<br>***1.630***	**1.245**<br>*0.216*
$T_4 \leftarrow T_1$	1.768<br>*2.025*	**0.638**<br>*0.144*	0.498<br>*0.891*	0.631<br>***0.992***	0.033<br>*0.599*	0.091<br>***1.051***	**1.226**<br>*0.376*
$T_5 \leftarrow T_1$	4.759<br>*6.386*	**2.087**<br>*0.557*	1.569<br>*0.839*	1.103<br>***4.990***	0.312<br>***2.628***	0.693<br>*1.921*	**3.753**<br>*1.836*
$T_6 \leftarrow T_1$	1.516<br>*1.288*	**0.888**<br>*0.158*	0.466<br>***1.004***	0.162<br>*0.126*	0.152<br>***0.701***	**1.233**<br>*0.401*	0.131<br>*0.184*

Table S6. Atomic group decomposition of the charge-transfer (CT) and spin-transfer (ST) excitation lengths, $\boldsymbol{D}_{CT}$ and $\boldsymbol{D}_{ST}$, for the first five singlet and triplet vertical excited states of TMTQ in nitromethane, computed using DFT and TD-DFT at the CAM-B3LYP/cc-pVDZ level. Only the z component, $\mathbf{d}^{\Omega}_{CT,z}$ (or $\mathbf{d}^{\Omega}_{ST,z}$), of each group contribution to the excitation length vector is reported, since the z axis is nearly collinear with $\boldsymbol{D}_{CT}$ and $\boldsymbol{D}_{ST}$ for most excitations. The degree of collinearity is described by $\alpha_{CT,z}$ (or $\alpha_{ST,z}$). Deviations of α from ±1 indicate the extent of departure from perfect collinearity (or anti-parallelism). The dimensionless quantities $\boldsymbol{g}^{\Omega}_{CT,z}$ (or $\boldsymbol{g}^{\Omega}_{ST,z}$) represent the ratio $\mathbf{d}^{\Omega}_{CT,z}/D_{CT}$ (or $\mathbf{d}^{\Omega}_{ST,z}/D_{ST}$). For each excitation, the largest absolute values of $\mathbf{d}^{\Omega}_{CT,z}$ and $\boldsymbol{g}^{\Omega}_{CT,z}$ (or $\mathbf{d}^{\Omega}_{ST,z}$ and $\boldsymbol{g}^{\Omega}_{ST,z}$) are highlighted in bold. All values are in Å, except for α and g which are dimensionless. Data were obtained with the DOCTRINE_SPIN code.

State	D_{CT}, (D_{ST})	$\boldsymbol{D}_{CT,z}$ ($\boldsymbol{D}_{ST,z}$)	$\alpha_{CT,z}$ ($\alpha_{ST,z}$)	$\mathbf{d}^{\Omega}_{CT,z}$, ($\mathbf{d}^{\Omega}_{ST,z}$) DCN	THIO	M10A	$\boldsymbol{g}^{\Omega}_{CT,z}$, ($\boldsymbol{g}^{\Omega}_{ST,z}$) [a] DCN	THIO	M10A
**Singlet states**									
$S_1 \leftarrow S_0$	0.021	0.021	0.982	-0.025	**0.060**	-0.014	-1.185	**2.848**	-0.681
$S_2 \leftarrow S_0$	0.099	0.098	0.992	-0.004	**0.091**	0.012	-0.043	**0.912**	0.122
$S_3 \leftarrow S_0$	0.151	0.151	0.999	0.036	**0.098**	0.016	0.241	**0.650**	0.108
$S_4 \leftarrow S_0$	0.235	-0.234	-0.996	-0.124	0.104	**-0.213**	-0.530	0.439	**-0.905**
$S_5 \leftarrow S_0$	0.043	0.026	0.604	-0.046	**0.181**	-0.110	-1.046	**4.181**	-2.531
**Triplet states: charge (*and spin*) values**									
$T_2 \leftarrow T_1$	0.520<br>*0.268*	-0.520<br>*0.258*	-1.000<br>*0.961*	-0.280<br>***0.193***	0.104<br>*-0.099*	**-0.343**<br>*0.164*	-0.540<br>***0.719***	0.200<br>*-0.369*	**-0.660**<br>*0.612*
$T_3 \leftarrow T_1$	0.855<br>*0.574*	-0.779<br>*0.523*	-0.911<br>*0.911*	**-0.461**<br>***0.416***	0.131<br>*-0.166*	-0.450<br>*0.274*	**-0.539**<br>***0.724***	0.154<br>*-0.290*	-0.526<br>*0.478*
$T_4 \leftarrow T_1$	0.917<br>*0.639*	-0.756<br>*0.466*	-0.824<br>*0.730*	**-0.445**<br>***0.325***	0.065<br>*-0.135*	-0.375<br>*0.276*	**-0.486**<br>***0.508***	0.072<br>*-0.212*	-0.410<br>*0.433*
$T_5 \leftarrow T_1$	1.192<br>*1.044*	-1.192<br>*1.044*	-1.000<br>*1.000*	-0.548<br>*0.449*	0.088<br>*-0.111*	**-0.732**<br>***0.705***	-0.459<br>*0.430*	0.074<br>*-0.106*	**-0.614**<br>***0.676***
$T_6 \leftarrow T_1$	0.666<br>*0.250*	-0.613<br>*0.243*	-0.922<br>*0.969*	**-0.739**<br>***0.612***	0.106<br>*-0.304*	0.020<br>*-0.065*	**-1.110**<br>***2.444***	0.159<br>*-1.216*	0.030<br>*-0.259*

(a) $\boldsymbol{g}^{\Omega}_{CT,z}$ (or $\boldsymbol{g}^{\Omega}_{ST,z}$) is a dimensionless quantity providing a measure of the $\mathbf{d}^{\Omega}_{CT,z}$ (or $\mathbf{d}^{\Omega}_{ST,z}$) length relative to the D_{CT} (or D_{ST}) length (see Eqs. 22a and 22b). A negative sign of $\boldsymbol{g}^{\Omega}_{CT,z}$ (or $\boldsymbol{g}^{\Omega}_{ST,z}$) means that $\mathbf{d}^{\Omega}_{CT,z}$ (or $\mathbf{d}^{\Omega}_{ST,z}$) is oppositely directed to $(\mathbf{R}^{+} - \mathbf{R}^{-})_z$ (or $(\mathbf{S}^{\uparrow} - \mathbf{S}^{\downarrow})_z$)

Table S7. Decomposition of the z-component of the charge-transfer (CT) and spin-transfer (ST) dipole moment vectors, $\boldsymbol{\mu}_{CT}$ and $\boldsymbol{\mu}_{ST}$ (in Debye, D), into *intra*-subdomain (μ_{CT}^{intra} or μ_{ST}^{intra}) and *inter*-subdomain (μ_{CT}^{inter} or μ_{ST}^{inter}) contributions, according to Eq. 25 (CT) and Eq. 26 (ST). Results refer to the first five singlet and triplet vertical excited states of TMTQ in nitromethane, computed using DFT and TD-DFT at the CAM-B3LYP/cc-pVDZ level. Only the z-component is reported, since the x and y components are negligible for most (though not all) excited states. Intra- and inter-subdomain contributions to $\boldsymbol{\mu}_{CT}$ ($\boldsymbol{\mu}_{ST}$) are derived from the $M_{CT}^{z,+}$ or $M_{CT}^{z,-}$ matrices (and from the $M_{ST}^{z,\uparrow}$ or $M_{ST}^{z,\downarrow}$ matrices), whose elements sum to $\boldsymbol{\mu}_{CT,z}^{+}$ and $\boldsymbol{\mu}_{CT,z}^{-}$ (or $\boldsymbol{\mu}_{ST,z}^{\uparrow}$ and $\boldsymbol{\mu}_{ST,z}^{\downarrow}$), respectively (Eqs. 24a-b). All data in the table were obtained using the DOCTRINE_SPIN code.

State	$\boldsymbol{\mu}_{CT,z}$<br>$\boldsymbol{\mu}_{ST,z}$	$\boldsymbol{\mu}_{CT,z}^{intra,+}$<br>$\boldsymbol{\mu}_{ST,z}^{intra,\uparrow}$	$\boldsymbol{\mu}_{CT,z}^{inter,+}$<br>$\boldsymbol{\mu}_{ST,z}^{inter,\uparrow}$	$\boldsymbol{\mu}_{CT,z}^{intra,-}$<br>$\boldsymbol{\mu}_{ST,z}^{intra,\downarrow}$	$\boldsymbol{\mu}_{CT,z}^{inter,-}$<br>$\boldsymbol{\mu}_{ST,z}^{inter,\downarrow}$
**Singlet states** [a]					
$S_1 \leftarrow S_0$	0.049	0.013 (26.5)	0.036 (73.5)	0.003 (5.8)	0.047 (94.2)
$S_2 \leftarrow S_0$	0.181	0.056 (30.9)	0.125 (69.1)	0.047 (26.0)	0.134 (74.0)
$S_3 \leftarrow S_0$	0.349	0.076 (22.1)	0.272 (77.9)	0.070 (20.1)	0.279 (79.9)
$S_4 \leftarrow S_0$	-0.893	-0.279 (31.2)	-0.614 (68.8)	-0.662 (74.1)	-0.230 (25.9)
$S_5 \leftarrow S_0$	0.057	0.013 (23.4)	0.043 (76.6)	-0.051 (-89.5)	0.108 (189.5)
**Triplet states: charge (*and spin*) values** [a]					
$T_2 \leftarrow T_1$	-1.250<br>*1.178*	-0.407 (32.6)<br>*0.360 (30.6)*	-0.843 (67.4)<br>*0.818 (69.4)*	-0.643 (51.4)<br>*0.256 (21.7)*	-0.606 (48.6)<br>*0.922 (78.3)*
$T_3 \leftarrow T_1$	-2.069<br>*2.687*	-0.301 (14.5)<br>*0.374 (13.9)*	-1.768 (85.5)<br>*2.313 (86.1)*	-0.657 (31.8)<br>*0.268 (10.0)*	-1.412 (68.2)<br>*2.419 (90.0)*
$T_4 \leftarrow T_1$	-1.457<br>*1.478*	-0.402 (27.6)<br>*0.372 (25.2)*	-1.055 (72.4)<br>*1.106 (74.8)*	-0.523 (35.9)<br>*0.211 (14.2)*	-0.934 (64.1)<br>*1.268 (85.8)*
$T_5 \leftarrow T_1$	-4.755<br>*6.385*	-1.098 (23.1)<br>*3.447 (54.0)*	-3.657 (76.9)<br>*2.938 (46.0)*	-2.351 (49.4)<br>*1.704 (26.7)*	-2.403 (50.6)<br>*4.681 (73.3)*
$T_6 \leftarrow T_1$	-1.397<br>*1.247*	-0.475 (34.0)<br>*-0.463 (-37.1)*	-0.922 (66.0)<br>*1.710 (137.1)*	0.016 (-1.1)<br>*0.588 (47.1)*	-1.413 (101.1)<br>*0.660 (52.9)*

a) In parentheses, the percentage values relative to the associated $\boldsymbol{\mu}_{CT,z}$ (or $\boldsymbol{\mu}_{ST,z}$) values are reported.

Table S8. Matrix elements (in Debye, D) of $M_{CT}^{z,+}$ and $M_{CT}^{z,-}$ (or $M_{ST}^{z,\uparrow}$ and $M_{ST}^{z,\downarrow}$) for the T_5 vertical excited state of TMTQ in nitromethane, computed using DFT and TD-DFT at the CAM-B3LYP/cc-pVDZ level. Matrix elements for the *x* and *y* components of the $\boldsymbol{\mu}_{CT}$ and $\boldsymbol{\mu}_{ST}$ vectors are omitted as their magnitudes are much smaller than those of the *z* component. Diagonal elements (pink) correspond to *intra-subdomain* contributions, $\boldsymbol{\mu}_{CT,z}^{intra,+}$, $\boldsymbol{\mu}_{CT,z}^{intra,-}$, $\boldsymbol{\mu}_{ST,z}^{intra,\uparrow}$, $\boldsymbol{\mu}_{ST,z}^{intra,\downarrow}$; off-diagonal elements (light blue) correspond to *inter-subdomain* contributions to $\boldsymbol{\mu}_{CT,z}^{+}$, $\boldsymbol{\mu}_{CT,z}^{-}$, $\boldsymbol{\mu}_{ST,z}^{\uparrow}$, $\boldsymbol{\mu}_{ST,z}^{\downarrow}$, respectively (see Eqs. 25-26)

$\boldsymbol{M_{CT}^{z,+}}$ **matrix for $T_5 \leftarrow T_1$ (values in Debye)**					
	$q_{CT}^{+}(DCN1)$	q_{CT}^{+}(DCN2)	q_{CT}^{+}(THIO1)	q_{CT}^{+}(THIO2)	q_{CT}^{+}(M10A)
$\mathbf{d}_{CT,z}^{DCN1}$	*-0.237*	-0.240	-0.178	-0.181	-0.252
$\mathbf{d}_{CT,z}^{DCN2}$	-0.239	*-0.242*	-0.180	-0.182	-0.254
$\mathbf{d}_{CT,z}^{THIO1}$	0.038	0.039	*0.029*	0.029	0.041
$\mathbf{d}_{CT,z}^{THIO2}$	0.038	0.039	0.029	*0.029*	0.041
$\mathbf{d}_{CT,z}^{M10A}$	-0.636	-0.645	-0.479	-0.485	*-0.678*
$\boldsymbol{M_{CT}^{z,-}}$ **matrix for $T_5 \leftarrow T_1$ (values in Debye)**					
	$q_{CT}^{-}(DCN1)$	q_{CT}^{-}(DCN2)	q_{CT}^{-}(THIO1)	q_{CT}^{-}(THIO2)	q_{CT}^{-}(M10A)
$\mathbf{d}_{CT,z}^{DCN1}$	-0.035	-0.036	-0.079	-0.079	-0.858
$\mathbf{d}_{CT,z}^{DCN2}$	-0.036	*-0.036*	-0.080	-0.080	-0.865
$\mathbf{d}_{CT,z}^{THIO1}$	0.006	0.006	*0.013*	0.013	0.138
$\mathbf{d}_{CT,z}^{THIO2}$	0.006	0.006	0.013	*0.013*	0.139
$\mathbf{d}_{CT,z}^{M10A}$	-0.095	-0.097	-0.213	-0.212	*-2.305*

$\boldsymbol{M_{ST}^{z,\uparrow}}$ **matrix for $T_5 \leftarrow T_1$ (values in Debye)**					
	$s_{ST}^{\uparrow}(DCN1)$	$s_{ST}^{\uparrow}$(DCN2)	$s_{ST}^{\uparrow}$(THIO1)	$s_{ST}^{\uparrow}$(THIO2)	$s_{ST}^{\uparrow}$(M10A)
$\mathbf{d}_{ST,z}^{DCN1}$	*0.059*	0.060	0.088	0.092	1.071
$\mathbf{d}_{ST,z}^{DCN2}$	0.060	*0.060*	0.088	0.093	1.075
$\mathbf{d}_{ST,z}^{THIO1}$	-0.015	-0.015	*-0.022*	-0.023	-0.265
$\mathbf{d}_{ST,z}^{THIO2}$	-0.015	-0.015	-0.022	*-0.023*	-0.264
$\mathbf{d}_{ST,z}^{M10A}$	0.187	0.189	0.277	*0.290*	*3.372*
$\boldsymbol{M_{ST}^{z,\downarrow}}$ **matrix for $T_5 \leftarrow T_1$ (values in Debye)**					
	$s_{ST}^{\downarrow}(DCN1)$	$s_{ST}^{\downarrow}$(DCN2)	$s_{ST}^{\downarrow}$(THIO1)	$s_{ST}^{\downarrow}$(THIO2)	$s_{ST}^{\downarrow}$(M10A)
$\mathbf{d}_{ST,z}^{DCN1}$	*0.281*	0.284	0.204	0.209	0.394
$\mathbf{d}_{ST,z}^{DCN2}$	0.282	*0.285*	0.204	0.210	0.395
$\mathbf{d}_{ST,z}^{THIO1}$	-0.070	-0.070	*-0.050*	-0.052	-0.098
$\mathbf{d}_{ST,z}^{THIO2}$	-0.069	-0.070	-0.050	*-0.051*	-0.097
$\mathbf{d}_{ST,z}^{M10A}$	0.883	0.893	0.641	0.658	*1.241*
